# Shifting phytoplankton ecological strategies along a continuum of tidewater glacier retreat

**DOI:** 10.1093/ismeco/ycaf045

**Published:** 2024-03-18

**Authors:** Patrick L White, Erin M Bertrand, Jenifer S Spence, Maria A Cavaco, Claire Parrott, Stephanie Waterman, Elden Rowland, Megan E Roberts, Terry Noah, Travis Mellett, Danielle Hallé, Andrew K Hamilton, Randelle M Bundy, David Didier, Maya P Bhatia

**Affiliations:** Department of Earth and Atmospheric Sciences, University of Alberta, Edmonton, AB T6G 2G5, Canada; Department of Biology, Dalhousie University, Halifax, NS B3H 4R2, Canada; Department of Biology, Dalhousie University, Halifax, NS B3H 4R2, Canada; Department of Earth and Atmospheric Sciences, University of Alberta, Edmonton, AB T6G 2G5, Canada; Department of Earth and Atmospheric Sciences, University of Alberta, Edmonton, AB T6G 2G5, Canada; Department of Earth, Ocean and Atmospheric Sciences, University of British Columbia, Vancouver, BC V6T 1Z4, Canada; Department of Earth, Ocean and Atmospheric Sciences, University of British Columbia, Vancouver, BC V6T 1Z4, Canada; Department of Biology, Dalhousie University, Halifax, NS B3H 4R2, Canada; Department of Biology, Dalhousie University, Halifax, NS B3H 4R2, Canada; Ausuittuq Adventures, Grise Fiord, NU X0A 0J0, Canada; School of Oceanography, University of Washington, Seattle, WA 98195, United States; Department of Geography and Environmental Management, University of Waterloo, Waterloo ON N2L 3G1, Canada; Department of Earth and Atmospheric Sciences, University of Alberta, Edmonton, AB T6G 2G5, Canada; School of Oceanography, University of Washington, Seattle, WA 98195, United States; Northern and Arctic coastal research lab, Center for Northern studies, Québec-Océan, Université du Québec à Rimouski, Rimouski, QC, G5L 3A1, Canada; Department of Earth and Atmospheric Sciences, University of Alberta, Edmonton, AB T6G 2G5, Canada

**Keywords:** primary productivity, marine-terminating glacier, ocean, biogeochemistry, Arctic, Rubisco, metaproteomics, trophic strategies, climate change, diatom

## Abstract

Marine-terminating (i.e., tidewater) glaciers are experiencing rapid retreat. Compared to land-terminating glaciers, tidewater glaciers can entrain nutrient-rich deep seawater with buoyant glacial meltwater released at depth from the glacier terminus, fueling summertime primary productivity. We used a continuum of tidewater glaciers at various stages of retreat in the Canadian Arctic Archipelago, in Inuit Nunangat, as a natural laboratory for approximating the impacts of tidewater glacier retreat on marine primary producers and their ecological strategies. We measured phytoplankton community composition and estimated productivity along this retreat continuum and found that phytoplankton communities consist mostly of fast-growing r-strategists such as diatoms at sites with tidewater glaciers likely to be capable of deep-water nutrient upwelling. At sites without tidewater glaciers or those with tidewater glaciers that may have retreated too much to upwell deep-water nutrients, we found communities dominated by small and potentially mixotrophic flagellates, which were indicative of regenerative production and low-nutrient environments. We also observed the highest estimated diatom carbon fixation potential co-occurring with chemical signals of upwelling near a shallow tidewater glacier. These finding suggest that shoaling tidewater glaciers can be important regions of summertime productivity when they can facilitate deep-water nutrient upwelling. However, with continued retreat, tidewater glaciers will cease deep-water upwelling. Low contributions of diatoms at sites with glaciers that no longer induce deep-water upwelling show that tidewater glacier shoaling will ultimately result in reduced ecosystem productivity and shifts towards phytoplankton that employ ecological strategies for success in stratified, nutrient-poor environments, with implications for marine ecosystems adjacent to the >1000 retreating Arctic tidewater glaciers.

## Introduction

Studies of Arctic glacier–ocean systems have found elevated nutrient concentrations near the surface of the ocean adjacent to marine-terminating (tidewater) glaciers [1–3]. These nutrients can originate from (i) glacial meltwater itself, which is often enriched in iron, manganese, and silica [4–9], and (ii) deeper marine waters, with elevated concentrations of nitrate, phosphate, and silicate delivered to surface waters via entrainment and upwelling associated with the surfacing of buoyant meltwater released to the ocean from the glacier terminus at depth [1, 2, 8]. Compared to Arctic coastal sites with land-terminating glaciers, sites near marine-terminating glaciers have also often been found to be post–spring bloom hotspots of summertime phytoplankton productivity [3, 10], as well as hotspots for higher–trophic level organisms like seals and birds [8, 11, 12]. However, physical factors like catchment bathymetry, bedrock lithology, oceanographic setting, and glacier morphology likely play important roles in the degree to which a tidewater glacier can mix the water column, influence near-surface nutrient concentrations, and stimulate phytoplankton growth [5, 8, 13]. Most of our understanding on the impacts of glacier upwelling on the Arctic marine environment comes from studies of tidewater glaciers interacting with coastal waters off Greenland [1–3, 14], but recent studies of tidewater glaciers in Canada [5, 10, 15] and on Svalbard [13, 16] have shown that tidewater glaciers in many Arctic regions may be enhancing local near-surface ocean nutrient concentrations and summertime marine productivity.

Studies of tidewater glacier–ocean systems involving glaciers with deep grounding lines (>150-m depth) in Greenland have started to show an association of diatoms with tidewater glaciers and meltwater-induced upwelling [14, 17–19] and smaller picoplankton with land-terminating glaciers [17, 18, 20]. Maselli *et al.* [17] also found differences in plankton trophic structure (e.g. the relative proportion of autotrophs, heterotrophs, and mixotrophs), with a higher proportion of autotrophs found near sites influenced by tidewater glaciers and a higher proportion of heterotrophs at sites without tidewater glacier influence. Several of the studies cited above [14, 17, 18] have linked the observed high abundances of diatoms with the potential upwelling of macronutrients. Furthermore, there have been many studies of phytoplankton community composition at tidewater glaciers grounded in shallow waters (grounding lines <100 m depth) in Svalbard (e.g. [13, 21–25]), but geographic factors complicate the relation of glacier-driven nutrient delivery with phytoplankton productivity and ecological strategies in that region [13]. Recently, Roberts *et al.* [10] showed that diatom Rubisco (i.e. the carbon fixation protein Ribulose-1,5-bisphosphate carboxylase/oxygenase), a key enzyme in carbon fixation that can serve as a proxy for primary production potential [26], was typically higher in abundance at tidewater glacierized sites than at nonglacierized sites in the Canadian Arctic Archipelago. However, Roberts *et al.* [10] did not compare Rubisco concentrations at tidewater glaciers of different sizes and grounding-line depths. These researchers also did not investigate any patterns in the expected ecological strategies employed by phytoplankton groups in response to glacier-driven water column changes in this system. Ecological strategies, such as the balance between mixotrophy and autotrophy and between regenerative and new production, have important consequences to be considered to further our understanding of the fate of carbon in ecosystems [27], the efficiency of trophic transfer, and niche partitioning [28]. Such consideration leads us to important unanswered questions about how phytoplankton communities change their composition and ecological strategies as glaciers transition from deep to shallow and to land terminating, and what impacts these transitions might have on marine primary productivity and ecosystems.

The Canadian Arctic Archipelago, located in Inuit Nunangat, is an ideal natural laboratory to examine how the retreat of tidewater glaciers may cause shifts in marine phytoplankton communities. These shifts occurring at the base of marine food webs in Inuit Nunangat could have important implications for Inuit communities, including the Hamlet of Grise Fiord (Ausuittuq), whose inhabitants utilize Jones Sound, a glacier-rich region of the Canadian Arctic Archipelago, for their harvests of marine animals such as fish, seal, and narwhal [29]. At present, tidewater glacier-hosting fjords and bays are important sites for marine production (Jimmy Qaapik personal communication) and there is concern that changing tidewater glaciers in this region could have negative consequences for higher trophic-level organisms and the communities that rely on them. Bhatia *et al.* [5] investigated potential impacts of tidewater glacier retreat on marine biogeochemistry by using a continuum of glacier systems ranging from deep to shallow tidewater glaciers and sites with no tidewater glaciers in Jones Sound. From data collected in 2019 in Jones Sound, Bhatia *et al.* [5] showed that deep tidewater glaciers can enhance surface ocean nutrient concentrations in the Canadian Arctic Archipelago, observations similar to those reported near Svalbard [13, 16] and Greenland [9, 30, 31]. These investigators hypothesized that so long as glacier meltwater is delivered to the ocean sufficiently below the nutricline at the tidewater glacier terminus, a tidewater glacier has the potential to stimulate phytoplankton through the delivery of nutrient-rich deep water to the surface.

In the investigation reported here, we aimed to expand on the study of Bhatia *et al.* [5] by examining how tidewater glacier retreat is likely to impact marine phytoplankton community structure, ecological strategies, and primary productivity during the summer peak glacier melt season. Throughout the Arctic, these summer months are marked by low nitrate availability post–spring bloom, where small plankton (K-strategists, those that are slow growing and tend to dominate in systems with high nutrient recycling rates and are often mixotrophic [32]) thrive [33–35]. We hypothesized that glaciers that drive delivery of deeper seawater, containing elevated nutrients, to the surface ocean cause a shift in summertime phytoplankton communities and their dominant ecological strategies that favors fast-growing phytoplankton, like diatoms, which dominate high-nutrient environments. To test our hypothesis, we characterized the phytoplankton community composition and estimated productivity in the near-termini regions on a continuum of tidewater glacier–ocean systems of varying grounding-line depths (between ~1 and 300 m), sampling as close as 150 m from the ice fronts. We then compared these results to those from sites that lacked tidewater glaciers entirely. This comparison showed that, generally, tidewater glaciers support a higher proportion of diatoms. However, some shallow tidewater glaciers that we hypothesized were unlikely to induce upwelling of deep-water nutrients supported phytoplankton communities that more closely resembled regions without tidewater glaciers, that is, communities dominated by smaller, flagellated, and potentially mixotrophic plankton. Additionally, we present evidence for a link between glacier-driven nutrient upwelling and enhanced diatom primary productivity. Our results demonstrate that tidewater glaciers of diverse sizes and with diverse grounding-line depths can fuel summertime productivity in Inuit Nunangat, but also suggest that ultimately a likely consequence of tidewater glacier retreat will be a reduction in diatoms and the summertime glacier-driven enhancement of marine productivity.

## Materials and methods

### Classifying tidewater glacier potential upwelling and tidewater glacier influence

**Figure 1:**
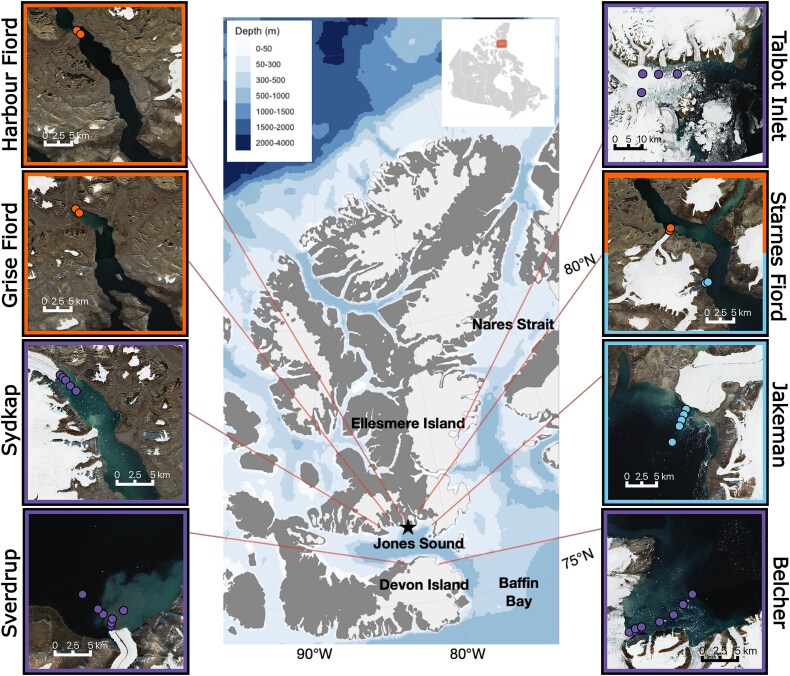
Map of stations across Jones sound and Nares Strait in Inuit Nunangat (Nunavut, Canada), with community of Ausuittuq (denoted by star). Insets show locations of bottle stations (coloured circles) at eight sites which include two sites without tidewater glaciers (Grise fiord, Harbour fiord), three sites we classified as under weak tidewater glacier influence (Starnes.1, Starnes.2, Jakeman), and four sites we classified as under strong tidewater glacier influence (Belcher, Sverdrup, Sydkap, Talbot Inlet). Center map was created with the R package ggOceanMaps [37] where glacier and land polygons are made with Natural Earth and bathymetry is created with IBCAO data [38], while insets were created in QGIS (v.3.30.0) using Copernicus sentinel data from august 2019 processed by Sentinel Hub.

In this study, we sampled 35 stations across 9 sites during the summer month of August, 2021 (Fig. 1 and Fig. S1) on board the sailing yacht (*S/Y) Vagabond* and a community-owned/operated vessel (*Ausuittuq Adventures*) from the Hamlet of Grise Fiord (Ausuittuq). We sampled glacierized and non-glacierized regions in Jones Sound and Nares Strait, two of the three major waterways of the Canadian Arctic Archipelago. A description of sampling sites can be found in the Supplemental Methods and Bhatia *et al.* [5]. We classified sites as regions with no tidewater glaciers and regions with tidewater glaciers that we hypothesized were unlikely to be able to upwell deep-water nutrients (i.e. were under weak tidewater influence) and those that were likely able to upwell deep-water nutrients (i.e. under strong tidewater glacier influence). We focused on the ability of a glacier to induce deep-water nutrient upwelling because of the extreme nitrogen limitation exhibited across the Arctic during these summer months [36] (but see the Discussion). We characterized glaciers as having the potential to cause upwelling of deep-water nutrients primarily based on observational evidence of upwelling in the present study or the study by Bhatia *et al.* [5], which includes chemical signatures (e.g., macronutrients and low levels of dissolved oxygen in the surface ocean near the glacier) and physical signals (e.g. 1026-isopycnal sloping towards the glacier, turbidity, and cold water extending from the deep to the surface near the glacier). We classified the Sverdrup, Belcher, Talbot Inlet, and Sydkap Glaciers as glaciers that can likely induce upwelling of deep-water nutrients (i.e. under strong tidewater glacier influence), and classified the Starnes.1, and Starnes.2 (Fig. S2), and Jakeman Glaciers as glaciers that are unlikely to cause upwelling of deep-water nutrients (i.e. under weak tidewater glacier influence) with the reasoning and caveats behind our tidewater glacier classification system found in Table S1.

### Water column sampling and processing

Bottle samples were generally collected from the near surface (5-m depth), the subsurface chlorophyll maximum (SCM; 20–40-m depth), or at 20-m depth in the absence of an SCM. On board the *S/Y Vagabond* sailing vessel, bottle samples were collected with a 10-l model 1080 (nonmetallic) GO-FLO sampling bottle. On the *Ausuittuq Adventures* vessel, 1.7-l Niskin model 1010 and 2.5-l GO-FLO model 1080 (nonmetallic) bottles were used. All GO-FLO and Niskin bottles were cleaned prior to the field according to seawater trace metal clean procedure [39]. Frozen samples (i.e. nutrients, DNA, and proteins) were frozen immediately on board the Vagabond. On board the Ausuittuq Adventures vessel, samples were kept cold in the dark and then frozen after being returned to our temporary laboratory in Ausuittuq within 2–10 hours of collection. The procedures for conductivity, temperature, and depth (CTD), nutrient, and trace-metal sampling were performed as described in Bhatia *et al.* [5], except that in 2021, metal samples were syringe filtered with 0.2-μm polytetrafluoroethylene (PTFE) filters (Cole-Palmer) upon collection in order to measure dissolved trace metals compared to the measured total dissolvable metals reported in Bhatia *et al.* [5].

### Dissolved trace metals

Acidified dissolved trace metal samples were analysed on a high-resolution inductively coupled plasma mass spectrometer (HR-ICP-MS) to determine concentrations of dissolved Fe, Mn, and Cd*.* Sample pre-concentrations were measured on an automated seaFAST-pico system [40]. Samples (10 ml) were buffered in-line to a pH of 6.2 ± 0.2 with a 5.4-M ammonia acetate buffer before being loaded onto a Nobias P1 resin. Samples were eluted with 1 N nitric acid spiked with 1 ppb indium as an internal standard. Sample eluents were measured on a high-resolution inductively coupled plasma mass spectrometer (Element XR; Thermo Scientific) and quantified by standard addition following internal standard correction with the blanks and references listed in Table S2.

### DNA and proteins

We filtered 4–7 l of water for protein samples and 1–2 l of water for DNA. Volumes filtered varied due to variations in particle loads. Protein samples at Sverdrup Glacier were taken in duplicate at each depth because the high biomass led to rapid filter clogging. Sampling and processing methodology for DNA and proteins were identical to those reported by Roberts *et al.* [10], except for slight modifications to protein extraction and purification described below. Sterivex™ filters (Fisher Scientific) were cut similarly to those described by Cruand *et al.* [41]. After digestion, reduction, and alkylation, proteins were desalted by suspending digested protein in 100 μL of 3% acetonitrile and 0.1% formic acid, loaded onto 50-mg C18 columns, and washed 3 times with 1-ml 0.1% trifluoroacetic acid. Peptides were further washed and then eluted from the column with 200 μl of 3% acetonitrile and 0.1% formic acid followed by 100 μl of 70% acetonitrile and 0.1% formic acid. Desalted samples were dried in a vacufuge (Eppendorf) for 2–4 hours (V-AQ setting). Peptides of interest were chosen based on taxonomic specificity as described in Roberts *et al.* [10] and Table S3. Justification and calculation of Rubisco-based productivity estimates can be found in Roberts *et al.* [10] and Young *et al.* [26].

To calculate carbon fixation from Rubisco concentrations, we used the following equation as discussed in detail in Roberts *et al.* [10]:


\begin{align*} & Carbon\kern0.17em fixation\kern0.17em rate\;\left(\frac{mg\;C}{m^3\times day}\right)\\ &\quad= RbcL\;\left(\frac{mol}{L}\right)\times{k}_{cat}^c\;\left(\frac{mol\;C}{mol\; active\kern0.17em site\times s}\right)\times \frac{1\; mol\; active\kern0.17em site}{1\; mol\; RbcL}\\ &\quad\quad\times \frac{12.011g\;C}{mol\;C}\times \frac{1000\;L}{m^3}\times \frac{86400\;s}{day} \end{align*}

Details on DNA extractions, amplicon sequencing, and bioinformatics processing were the same as those found in Roberts *et al.* [10]. In brief, the V4–V5 hypervariable region of the 16S rRNA gene was amplified using the 515F/926R primers [42]. The SILVA v.138 (2019) reference database was used to assign taxonomic annotations to amplicon sequence variants (ASVs) using the QIIME 2 v.2022.2.0 pipeline [43]. All ASVs assigned to chloroplasts (5% of ASVs, average 6.4% of total reads) were further assigned to photosynthetic eukaryotic phytoplankton using PhytoREF, with the recognition that PhytoREF lacks sufficient representative sequences of dinoflagellates [44]. As the focus of this investigation was on single-celled phytoplankton, five ASVs were removed from the class of multicellular brown algae *Phaeophyceae* and 12 ASVs were removed that were assigned to land plants.

### Data processing and analysis

CTD data were processed and binned at intervals of 0.5 m in MATLAB (v.2022; Mathworks, Natick, MA, USA). Buoyancy frequency (N^2^) was calculated with the GSW toolbox [45] using a 10-point (5-m) running average. When we investigated the physical and chemical conditions at the discrete bottle sample depths, CTD data were averaged over 5-m intervals centered on the bottle target sampling depths. This choice was deemed necessary to account for uncertainty in the actual bottle sampling depths resulting from the line angle, which was difficult to avoid given our small vessel sampling setup and represents an attempt to best characterize water properties at the actual depths of the bottle samples despite large vertical gradients in water properties that can be common in glacier-influenced coastal waters.

`Data analysis was conducted in R (v4.4.2) [46] using the following packages: *plyr* [47]*, tidyverse* [48]*, ggplot2* [49]*, dplyr* [50]*, magrittr* [51]*, data.table* [52]*, ape* [53]*, gridExtra* [54]*, ggpubr* [55], *stringi, cowplot* [56]*,* and *vegan* [57]. For the principal component analysis (PCA) and distance-based redundancy analysis (dbRDA), water column physical and chemical data were scaled around the mean prior to calculating the Euclidean distances. Variables selected as significant by the forwards-backwards stepwise dbRDA were checked for multicollinearity (>10). Samples missing at least one physical and/or chemical water column variable (*n* = 5 of 75) were excluded from the PCA (*n* = 12 of 75) and dbRDA (*n* = 11 of 71). Raw data were shown instead of boxplots for groups with fewer than five observations. All nutrient values, including those below the limits of detection and quantification, were used as generated by the instrument for analysis [58]. Relative abundance was calculated from the raw reads for taxonomic bar plots, and read counts were Hellinger transformed prior to statistical testing and ordination by non-metric multidimensional scaling (NMDS). An analysis of similarities (ANOSIM) was performed to assess statistical differences between phytoplankton community composition between sites grouped as under either weak or strong tidewater glacier influence. The Wilcoxon rank-sum test was used to evaluate differences between strong tidewater glacier and weak tidewater glacier chemical and physical variables. We did not include the no-tidewater glacier group in the statistical comparison due to the small sample size.

## Results

### Physical and chemical variability across a continuum of tidewater glacier influence

**Figure 2:**
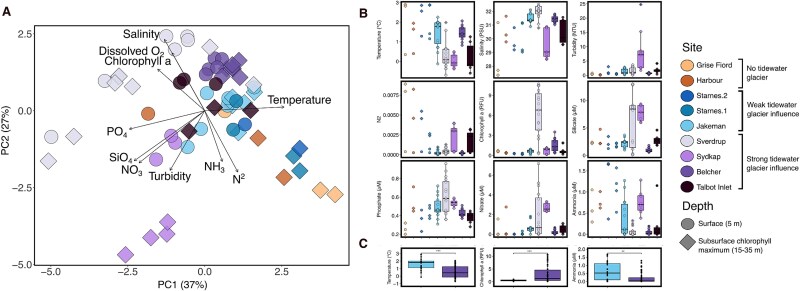
(A) PCA of physical (N^2^, turbidity, temperature, dissolved O_2_, chlorophyll *a*) and chemical (NO_3_^−^, PO_4_^3−^, NH_3_, SiO_4_) water column variables. Shapes represent samples taken from either the surface (5 m) or SCM (15–35 m) depth (see legend). (B) Boxplots of temperature (°C), salinity (PSU), turbidity (NTU), N^2^, dissolved oxygen (μM), chlorophyll *a* (relative fluorescence units, RFU), silicate (μM), phosphate (μM), nitrate (μM), and ammonia (μM) across sites. Sample depths range between 5–35-m. points indicate individual sample values, the black horizontal lines indicate median value, upper and lower limits of the box represent the 25th and 75th interquartile range, and the whiskers extend up to 1.5× the interquartile range. Sites with five or less values show the raw data instead of boxplots. (C) Boxplots of variables from (B) which were significantly different (Wilcoxon rank sum, ^*^P < .05, ^*^^*^P < .01 and, ^*^^*^^*^P < .001) between samples from strong tidewater glacier influence and weak tidewater glacier influence. Stations without tidewater glaciers were not compared in statistical analysis due to the small size of the group. See Methods for classification of tidewater glacier influence.

We used a PCA to describe multivariate patterns in the water column found across our tidewater glacier continuum to provide context for our phytoplankton-focused analyses (Fig. 2A). Values for each site and variable are shown as box plots in Fig. 2B, and section plots in Figs. S3, S4, S5, S6, S7. Generally, sites without tidewater glaciers exhibited the highest stratification and had the highest ammonia concentrations (ammonia = 1.67 μM; Fig. 2A-B). Similarly, sites classified as under weak tidewater glacier influence saw significantly increased ammonia concentrations (Fig. 2C) and a trend of elevated stratification (Fig. 2A). In comparison, sites that we classified as under strong tidewater glacier influence generally had significantly lower ammonia and higher chlorophyll *a* fluorescence than weak tidewater-glacier influenced sites (Fig. 2C). Sites with weak tidewater glacier influence generally had significantly higher water temperatures (Fig. 2C). The highest concentrations of major bioessential macronutrients (nitrate = 4.95 μM, phosphate = 0.8 μM, silicate = 12.9 μM) and highest chlorophyll *a* fluorescence occurred at Sverdrup Glacier, a strong tidewater-glacier-influenced site. However, not all strong tidewater-glacier-influenced sites were characterized by high nutrient concentrations at the time of sampling. For example, stations along both the Talbot Inlet and Belcher Glacier transects had generally low macronutrient concentrations (Fig. 2A-B). These depleted nutrient concentrations are coincident with lower chlorophyll *a* fluorescence observed at both Talbot Inlet and Belcher Glacier stations, compared to those observed at Sverdrup Glacier stations. These elevated nutrients at Sverdrup may relate to potential upwelling observable from satellite (Fig. S8). Higher nutrient concentrations were not always associated with higher chlorophyll *a*; for example, stations at Sydkap Glacier had some of the highest average macronutrient concentrations (nitrate = 2.7 μM, phosphate = 0.5 μM, silicate = 7.5 μM) observed in this study, but also had persistently low mean chlorophyll *a* and were relatively stratified (Fig. 2A-B).

### Phytoplankton community composition

**Figure 3:**
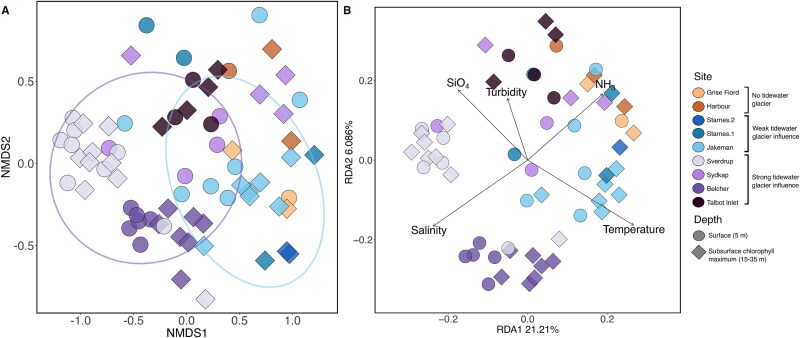
(A) Phytoplankton community composition utilizing an NMDS ordination (stress = 0.16). Ellipses represent 0.8 confidence intervals and denote groupings of samples into strong tidewater-glacier influence and weak tidewater-glacier influence, which are significantly different (ANOSIM, *R* = 0.38, *P* < .05). Stations without tidewater glaciers were not compared in statistical analysis due to the small size of the group. (B) Distance-based redundancy analysis (dbRDA) of the phytoplankton community composition with phosphate, nitrate, silicate, ammonia, turbidity, temperature, and salinity (adj. *R*^2^ = 25%). Vectors indicate significant physical and chemical variables (*P* < .05) as selected by our forwards–backwards selection. Shapes represent samples taken from either the surface (5-m) or SCM (15–35-m) depth. See methods for classification of tidewater glacier influence.

By using an NMDS to look for broad trends in phytoplankton community composition across stations, we saw there was a general separation between groups we classified as either under weak tidewater glacier influence or under strong tidewater glacier influence (ANOSIM, *R* = 0.38, *P*-value <.05; Fig. 3A). Furthermore, communities generally appeared to group by site. For example, communities at the Sverdrup, Belcher, Talbot Inlet, and Jakeman Glaciers generally clustered closely together within their respective sites. Both physical and chemical variables were identified as significant in describing the variance of the phytoplankton community composition through the dbRDA analysis, accounting for 25% of the variance (Fig. 3B).

**Figure 4:**
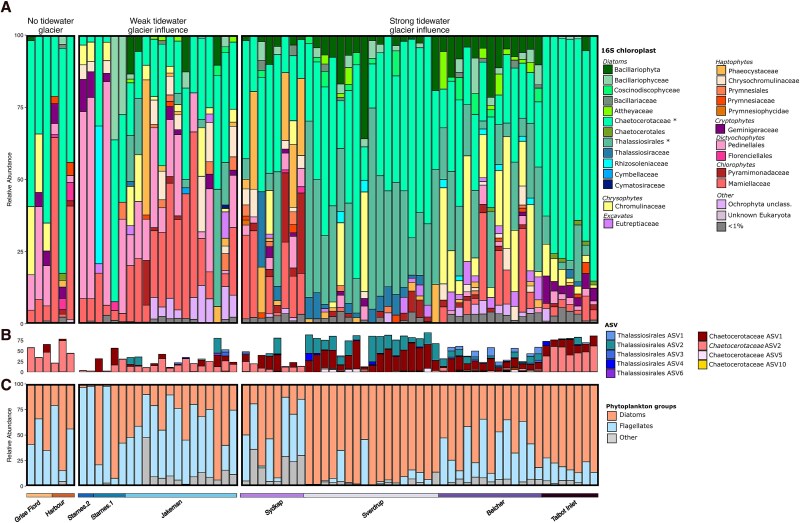
(A) The relative abundance of all phytoplankton taxonomic groups. Sample depths range between 5 and 35-m. Taxonomies were grouped to the lowest taxonomic resolution down to the family. Phytoplankton found at less than 1% relative abundance in any sample were categorized as <1%. (B) the relative abundance of the most abundant ASVs assigned as *Chaetocerotaceae* or *Thalassiosirales*, with additional ASVs shown in Fig. S9. (C) the relative distribution of flagellates and diatoms across sites. Many of the flagellates are identified as potential mixotrophs as described in the supplemental methods and in Table S4. Sites are grouped according to their classification of tidewater glacier influence, as shown by bottom color bars and the titles at the top. See the Methods for classification of tidewater glacier influence.

Diatoms contributed to a large proportion of the chloroplast 16S rRNA genes at the sites we classified as being under strong tidewater glacier influence, with a mean relative abundance across all strong tidewater-glacier–influenced stations of 72% (Fig. 4A–C). Groups such as *Attheyaceae*, *Rhizosoleniaceae*, and *Thalassiosiraceae* exhibited higher relative abundances at sites with strong tidewater glacier influence compared to those at sites with weak tidewater glacier influence or sites without tidewater glaciers. Within each strong tidewater-glacier–influenced site, distinct patterns of diatom abundances emerged. For example, *Thalassiosirales* was mostly abundant at the Belcher and Sverdrup Glaciers, whereas at the Talbot Inlet Glaciers, *Chaetocerotaceae* comprised a relatively high proportion of the phytoplankton community (x- = 72%). Generally, *Chaetocerotaceae* diatoms were universally present at all stations sampled, with a high average relative abundance of 36% of 16S chloroplast amplicons. Further investigation into patterns of *Chaetocerotaceae* ASVs and *Thalassiosirales* ASVs showed distinct groupings across our continuum of tidewater glacier influence and sites. For example, the “*Chaetocerotaceae* ASV1” exhibited a greater relative abundance at sites strongly influenced by tidewater glaciers and “*Thalassiosirales* ASV2” was more abundant at the Sverdrup Glacier, whereas “*Thalassiosirales* ASV1” was more abundant at the Belcher Glacier (Fig. 4B, Fig. S9). It should be noted that not all sites we classified as under strong tidewater glacier influence were dominated by diatoms. At Sydkap Glacier there was a high relative abundance of the haptophyte *Phaeocystaceae,* a family not seen as prominently at any other site regardless of the tidewater glacier continuum, along with a high relative abundance of the green algae family *Mamiellaceae* (Fig. 4A).

Flagellates appeared at sites spanning all degrees of our classification of tidewater glacier influence, but these organisms had a greater mean relative abundance at sites without a tidewater glacier and those with weak tidewater glacier influence, with flagellates comprising 53% of 16S chloroplast amplicons on average at these stations (Fig. 4C). This high relative abundance of flagellates was in large part the result of groups such as *Pedinellales* and *Chromulinaceae* being prolific at sites with weak tidewater glacier influence and sites without tidewater glaciers (Fig. 4A). In comparison, at sites with a strong tidewater glacier influence, flagellates had an average relative abundance of only 24%. Many of these flagellates are possible mixotrophs, as there is evidence from the literature that these organisms can acquire carbon through photosynthesis as well as through phagotrophy (Table S4).

### Rubisco across sites with strong tidewater glacier influence

**Figure 5:**
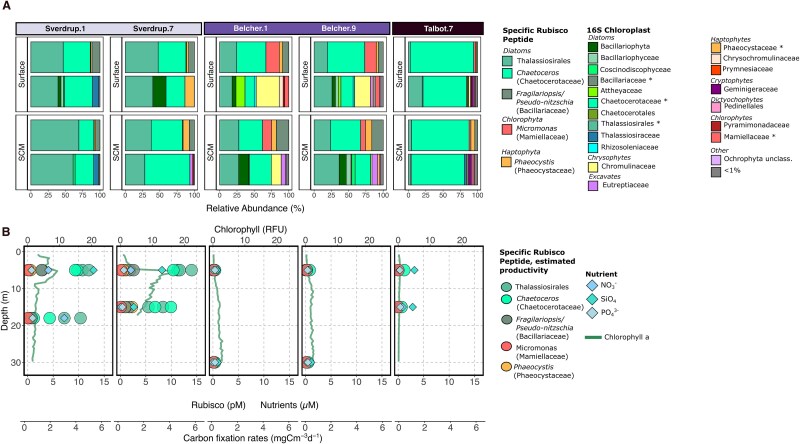
Rubisco concentrations from stations near three of the four sites we classified as under strong tidewater glacier influence: Sverdrup glacier, Belcher glacier, and Talbot inlet. Rubisco samples at Sverdrup were collected in duplicate at each depth (see Methods). (A) Bar plots showing relative phytoplankton community composition as determined by taxon-specific Rubisco quantification method (top) and 16S chloroplast rRNA gene amplicon sequencing (bottom) at the surface (5-m) and SCM (15–30-m) depths. Taxonomic naming convention for 16S chloroplast can be seen in Fig. 4. The taxonomies shared in 16S and Rubisco data are indicated by a ^*^. Relative Rubisco data was averaged at each Sverdrup station and depth where duplicates were taken (Methods). (B) Taxon-specific Rubisco concentrations and estimated carbon fixation rates [10] at the surface and SCM (circles), the station vertical profile of chlorophyll a fluorescence from the CTD sensor (green line), and macronutrient concentrations (NO_3_^−^ = nitrate, PO_4_^3−^ = phosphate, SiO_4_ = silicate) at the surface and SCM (diamonds). Sverdrup.1 has no silicate at the SCM. Full chlorophyll *a* profiles can be seen in Fig. S4.

The absolute and relative concentrations of group-specific Rubisco varied across select sites that we classified as being under strong tidewater glacier influence (Fig. 5A and B). These Rubisco measurements provide information about group-specific contributions to primary productivity. At the sites where Rubisco concentrations were measured (i.e. the Sverdrup, Belcher, and Talbot Inlet Glaciers), we expected that these Rubisco peptide measurements would capture a large proportion of the photosynthetic community. The relative abundances of Rubisco peptides likely originating from *Thalassiosirales*, *Chaetocerotaceae*, and *Mamiellaceae*, respectively, were similar to the abundances revealed by the 16S chloroplast–based community composition, respectively (Fig. 5A). These three groups (*Thalassiosirales*, *Chaetocerotaceae*, and *Mamiellaceae*) comprise 75% on average of the relative abundance of the 16S chloroplast data, suggesting that our Rubisco peptides are likely to be capturing the major photosynthetic groups identified in the 16S data.

Sverdrup Glacier stations had the highest concentrations of Rubisco associated with diatoms (Fig. 5B), and the sums of these diatom Rubisco concentrations were generally 10–20 times higher than concentrations at either the Belcher or Talbot Inlet Glaciers (Table S5). Diatom Rubisco concentrations generally increased with increasing macronutrient concentrations (Fig. 5B), and the sum of diatom Rubisco peptide concentrations exhibited a significant positive correlation with phosphate and silicate concentrations (Pearson *R* = 0.89 and 0.66 [*P* < .05] in both cases, Fig. S10 B and C) but not nitrate (Pearson R = 0.51, *P* < .05, Fig. S10A). The sum of diatom Rubisco peptide concentrations did not correlate significantly with any trace metal concentrations (Pearson *P* < .05, Fig. S10 D–F).

## Discussion

### Possible influences of tidewater glaciers on phytoplankton

In the Arctic, physical aspects of the water column, such as temperature, have been shown to impact community composition, for example with dominance of picoplankton typically associated with warmer waters [33, 34]. However, we also know that in the Arctic nutrients are important in shaping community composition [59] and primary production [38]. Here, both nutrients and physical variables such as temperature were significant in explaining the variance of the plankton community (Fig. 3B). Tidewater-glacier meltwater–induced upwelling can promote water column mixing and, depending on the glacier depth and the structure of the water column, could elevate cool water and mix the warm and stratified surface. However, only glaciers that terminate below the nutricline can also induce upwelling of deep-water nutrients. Importantly, variables such as temperature are conservative while nutrients, particularly limiting nutrients, are drawn down rapidly due to biological consumption, making assessment of the direct impact of enhanced nutrients on phytoplankton difficult without physiological measurements.

While temperatures were significantly lower at sites we classified as under strong tidewater glacier influence, ammonia concentrations were also significantly lower, possibly indicating that sites with a weak tidewater-glacier influence are dominated by recycling, whereas systems with a strong tidewater influence benefit from the input of new nutrients from deeper, colder water [32]. Undoubtedly temperature and other physical factors such as salinity are shaping the composition of phytoplankton communities across our sites. However, given the extreme nitrogen limitation across the Arctic Ocean during the summer [38] and the high nitrogen requirement of diatoms [60], we expect that the delivery of deep-water nutrients via tidewater glacier entrainment plays a crucial role in allowing diatoms to persist near tidewater glaciers during the summer months.

### Phytoplankton ecological strategies and tidewater glaciers

Compared to sites strongly influenced by tidewater glaciers, sites under weak tidewater-glacier influence appeared similar to those with no tidewater glaciers with respect to phytoplankton community composition: there was a reduced proportion of diatoms and an increased proportion of small phytoplankton groups that are known to thrive in low-nutrient environments, such as *Mamiellaceae*, *Pedinellales*, and *Chromulinaceae*, as a result of being small and slow growing. This trend may be the result of increased seasonal stratification in these Arctic fjords, limiting nutrient mixing of deeper marine waters [20]. We identified many of the flagellated plankton found at sites without tidewater glaciers and sites with weak tidewater-glacier influence to be potentially mixotrophic (Table S4), possibly utilizing phagotrophy to augment their nutritional requirements in these nutrient-limited environments. Generally, these smaller plankton are thought to support less trophic transfer compared to diatoms [32]. As a result of the differences in ecological strategies that we observed at the base of the marine food web across the sites we sampled, we hypothesize that sites without tidewater glaciers and sites under weak tidewater-glacier influence may provide less support to higher trophic animals (e.g., zooplankton, fish) compared to sites with strong tidewater-glacier influence, which is consistent with past findings [3, 8, 18]. This effect on higher trophic levels could impact local Inuit Nunangat communities that regularly harvest from waters influenced by tidewater glaciers, and further investigation is needed to better elucidate the holistic ecosystem impacts of these differences in phytoplankton strategies.

While we did see general trends in phytoplankton community composition across our degree of tidewater glacier influence, we also observed unique communities across some sites. For example, at Sydkap Glacier (i.e. one of the sites strongly influenced by tidewater glaciers), there was low abundance of diatoms and high abundance of the group *Phaeocystaceae* (Fig. 4A), likely *Phaeocystis*. Given that stations at Sydkap Glacier were particularly turbid (Fig. S4), and previous work has shown that *Phaeocystis* is better adapted than diatoms to niches where light levels are low and may fluctuate [61], these conditions may have given *Phaeocystis* an advantage in this turbid system. In contrast, we observed *Chaetocerotaceae* throughout all locations, regardless of tidewater-glacier influence. This finding is not surprising given that this group includes *Chaetoceros*, which is well documented to be ubiquitously present across the Arctic [62–64]. However, the distinct *Chaetocerotaceae* ASVs that were differentially abundant across the degrees of our tidewater glacier influence continuum (Fig. 4B) suggest the occurrence of multiple unique *Chaetocerotaceae* species occupying different ecological niches.

### Timing of glacial meltwater delivery matters

An important consideration when interpreting results in these tidewater glacier ecosystems is the timing of sampling. Kanna *et al.* [14] have shown that near tidewater glaciers in Greenland, diatom abundances, determined by microscopy, varied year-by-year, potentially owing to yearly differences in glacial melt volumes. The timing of meltwater delivery also appears to be important in the tidewater-glacier systems presented in this study. We used Sentinel 2 satellite imagery (Fig. S8) to evaluate the terminus region of the Sverdrup Glacier for visual indications of upwelling (e.g. turbid plume extending from the terminus). Such evaluation was possible only at Sverdrup Glacier because a previous investigation has identified where the subsurface plume exits [15], and our sampling days at this site coincided with cloud-free periods. Satellite imagery appears to show an upwelling event at Sverdrup glacier just before, or during, the time of sampling (Fig. S8). Previously identified water column signatures of meltwater-induced upwelling at these glaciers, including an upward sloping 1026 isopycnal and waters low in dissolved oxygen extending to the surface near the glacier [5], were also observed near Sverdrup Glacier in this study (Fig. S5). We also saw these signatures in the water column near Belcher Glacier, but they only extended to ~50 m below the surface, suggesting insufficient meltwater discharge for the upwelling to reach the surface at the time of sampling [65, 66]. This lack of upwelling to the surface at Belcher Glacier, and subsequent low levels of macronutrients, may be why some stations near Belcher Glacier had higher proportions of potentially mixotrophic groups like *Chromulinaceae* and *Pedinellales,* whereas phytoplankton at Sverdrup Glacier stations were almost entirely diatoms (Fig. 4, A and C). These results could also suggest that the relative differences in community composition (e.g., lack of diatoms) we observed in areas under weak tidewater glacier influence were simply the result of not sampling during an upwelling event. However, diatoms still made up a large proportion of the phytoplankton community composition at many Belcher and Talbot Inlet Glaciers stations (i.e. two sites we classified as under strong tidewater glacier influence), despite no clear evidence of upwelling to the surface at either of these glaciers during sampling. Generally, higher abundances of diatoms at sites strongly influenced by tidewater glaciers are consistent with higher frequencies of sampling of phytoplankton community composition from Greenland, which found diatoms to persist in a tidewater glacierized fjord throughout the summer, not just during times of potential peak meltwater delivery [18]. Together, these results suggest that periodic nutrient-rich deep-water upwelling to the surface at tidewater glaciers could promote a greater proportion of diatoms in the community throughout the summer, not just during active meltwater delivery. As such, we hypothesize that the lack of diatoms we observed at sites classified as under weak tidewater glacier influence is evidence of the inability of these weak tidewater-glacier–influenced systems to generate deep-water upwelling, rather than a mismatch between timing of sampling and upwelling.

A mismatch in timing is, however, important for quantifying the impact of meltwater-driven upwelling on primary productivity. The elevated macronutrients in the surface at Sverdrup Glacier (in part, a chemical signal for glacier upwelling) coincided with elevated diatom Rubisco protein concentrations (i.e. elevated potential for diatom primary productivity). This finding suggests that macronutrients, and especially nitrate, given its scarcity in the water column at this time of year [38], are likely key for enhancing marine primary production in these tidewater glacierized systems. While only silicate and phosphate, and not nitrate, were significantly positively correlated with diatom Rubisco (i.e. diatom productivity; Fig. S10, B and C), we suggest that this situation is due to the rapid consumption of the limiting nutrient nitrate, leaving excess phosphate and silicate in the water column (mean nitrate:phosphate:silicate is 3:1:10 at the Sverdrup Glacier). This low N:P:Si ratio provides evidence of rapid nitrate consumption and the importance of nitrate for diatoms in these tidewater glacierized systems, and also possibly explains the lack of correlation of nitrate concentrations with diatom productivity. As such, had we observed evidence for active upwelling and macronutrient delivery to the surface at the Belcher and Talbot Inlet Glaciers, we would have expected to have observed increased diatom Rubisco concentrations at these sites, similar to that observed at Sverdrup Glacier.

### Productivity at shallow tidewater glaciers provides insight into possible consequences of tidewater glacier retreat

Many investigations relating the impacts of tidewater glacier nutrient upwelling on phytoplankton community composition have occurred at glaciers with grounding-line depths >150-m [14, 17, 18], but ultimately, as tidewater glaciers retreat, they will have to become increasingly shallow until they are no longer in the ocean. While the timing of this retreat onto land will vary for different glaciers depending on various factors (e.g. glacier bed slope, speed of retreat, characteristics of the subglacial drainage network), the inevitability of tidewater glacier retreat suggests that determining the influence of shallow tidewater glaciers on productivity is critical for understanding what may happen as deeper glaciers shoal and eventually terminate on land. In this study, we looked at diatom productivity through Rubisco and found that diatom-specific Rubisco concentrations at the relatively shallow Sverdrup Glacier were an order of magnitude larger than concentrations at the much deeper Belcher and Talbot Inlet Glaciers. While the terminus depth of Sverdrup Glacier remains a point of contention, the depth of meltwater discharge is estimated to be ≥30-m [5, 15], which is likely hundred(s) of metres shallower than the Belcher and Talbot Inlet Glaciers (Table S1). As discussed, sampling during upwelling events is likely important for observing increased diatom productivity, meaning that higher diatom Rubisco concentrations observed at Sverdrup Glacier do not necessarily indicate that waters near Sverdrup Glacier are always more productive than those near the deeper Belcher and Talbot Inlet Glaciers. However, the elevated Rubisco at Sverdrup Glacier does show that shallow tidewater glaciers can be important regional hotspots of summertime productivity.

The other relatively shallow tidewater glaciers in this study, the Jakeman, Starnes.1, and Starnes.2 Glaciers (Table S1) did not appear as productive as the waters near Sverdrup Glacier. While we cannot compare diatom productivity directly across these sites, the 16S rRNA gene-based community composition at the Jakeman, Starnes.1, and Starnes.2 Glacier stations were dominated by small, flagellated, and likely mixotrophic plankton whereas Sverdrup communities were almost entirely diatoms (Fig. 4 A–C). These results could suggest that there are differences at Sverdrup Glacier that allow it to uniquely stimulate diatoms among the relatively shallow tidewater glaciers we sampled near in this study. Some researchers [5, 65] have suggested that the depth of glacial discharge in relation to the nitracline is likely important in determining whether an Arctic tidewater glacier can stimulate phytoplankton. In our study system, Jones Sound, the average nitracline depth was measured to be ~30-m deep [10], which may position the nitracline above the meltwater discharge depth of some shallow tidewater glaciers. The Starnes.2 Glacier is likely at the extreme of what can be classified as a tidewater glacier, where we estimate that only a small portion of the terminus resides in a few meters of water (Table S1; Fig. S2). The very high relative abundance of small, flagellated plankton at the Starnes.2 Glacier (x- = 93%) provides evidence that the extreme of tidewater glacier retreat may result in increased abundance of small and potentially mixotrophic plankton (Table S4). Furthermore, our estimated grounding line of the Starnes.1 Glacier is roughly 25-m, which may be too shallow to upwell deep-water nutrients (Table S1). In contrast to the Starnes Fiord glaciers, the Jakeman Glacier may extend deep enough to terminate below the nitracline (Table S1), but because much of the terminus of the Jakeman Glacier is no longer situated in the ocean, it is possible that the majority of meltwater is released on land first and not as submarine discharge [5]. A potential inability to upwell deep-water nutrients may be why we did not observe increases in diatom abundances at the Starnes.1, Starnes.2, or Jakeman Glaciers compared to nearly all of the other tidewater glaciers sampled nearby in this study. However, given our uncertainties with the terminus depth of the glaciers in this study, more insight into the depth of submarine discharge in relation to the nitracline would be needed to better constrain the relationship of meltwater-induced deep-water nutrient upwelling on marine phytoplankton. Additionally, more work, for example employing experimental and physiological approaches, is needed to better constrain the impacts of nutrients compared to impacts of other factors such as salinity and temperature. Still, the evidence we present here, and recent evidence of the importance of upwelled macronutrients for diatoms at deeper glaciers in Greenland [14, 18], suggests that upwelling of macronutrients may be key for enabling tidewater glaciers to enhance ecosystem productivity.

## Conclusions

Through looking across a continuum of tidewater glaciers, we show that relatively shallow tidewater glaciers can still be regions of enhanced productivity, but that likely only glaciers that can induce upwelling of deep-water nutrients can support a higher proportion of diatoms and lower proportion of flagellated and potentially mixotrophic plankton. These observvations suggest that potential consequences of tidewater glacier retreat may include reduction of diatom community contributions and reduction of productivity associated with these glacier–ocean systems. While the productivity we observe at a relatively shallow tidewater glacier suggests that tidewater glaciers may continue to stimulate productivity during retreat, inevitably, we expect that these once-productive summertime marine ecosystems may see shifts towards reduced productivity as glaciers retreat to shallow water and can no longer upwell deep-water nutrients.

## Supplementary Material

ismeC_02252025_supp_ycaf045

isme_supp_tables_02252025_ycaf045

## Data Availability

All data used in this study is freely and openly available. CTD and biogeochemical data can be found on Canadian Integrated Ocean Observing System (CIOOS) at https://doi.org/10.71708/zgvv-xk59 and https://doi.org/10.71708/3vbv-a128, respectively. DNA sequences were accessioned at NCBI (PRJNA905107). Additionally, all scripts and associated metadata for analysis can be found on the Bertrand Lab GitHub (https://github.com/bertrand-lab/CAA_PPCC.git).
