# Supplementary material for: Shifting phytoplankton ecological strategies along a continuum of tidewater glacier retreat
Source: ISME Commun. 2024 Mar 18;5(1):ycaf045. doi: 10.1093/ismeco/ycaf045 (PMC12011080; doi:10.1093/ismeco/ycaf045)
Supplement: ismeC_02252025_supp_ycaf045 [file ismec_02252025_supp_ycaf045.pdf]

Supplemental materials and methods

6.1. Descriptions of sampling and sampling sites

Water column sampling took place from August 2 to August 29, 2021, in the Canadian Arctic Archipelago, focusing on the marine regions of Jones Sound and Talbot Inlet (Nunavut, Canada). Sampling sites consisted of marine waters adjacent to seven tidewater glaciers (Sverdrup Glacier, Belcher Glacier, Sydkap Glacier, Talbot Inlet, Jakeman Glacier, Starnes.1 Glacier, Starnes.2 Glacier), as well as coastal waters at two sites without tidewater glaciers (Grise Fiord, Harbour Fiord; Figure 1). The oceanographic setting and description of Sverdrup Glacier, Belcher Glacier, Sydkap Glacier, Jakeman Glacier, and Grise Fiord can be found in Bhatia et al. [1]. We added three new sites in the current study not previously described in Bhatia et al.[1] : Starnes Fiord and Harbour Fiord located on Southern Ellesmere Island, and Talbot Inlet located on Eastern Ellesmere Island. Starnes Fiord is a relatively narrow fjord that forks at roughly 18 km inward, with each branch continuing to 32 km and 50 km in length respectively. Sampling in Starnes Fiord occurred along the longer branch of the fiord at, to our knowledge, two unstudied glaciers referred to herein as Starnes.1 Glacier and Starnes.2 Glacier. Starnes.1 and Starnes.2 Glaciers are relatively small tidewater glaciers with by far the smallest estimated summer discharge of all the glaciers in this study (Table S1). Harbour Fiord is a 30 km long fjord with a land-terminating glacier that drains into the head of the fjord approximately 8 km from shore. Lastly, Talbot Inlet contains many tidewater glaciers including Trinity and Wykeham Glaciers, the two largest (3,086 km^2 combined catchment) and fastest flowing glaciers in the Canadian Arctic. These glaciers drain the Prince of Wales icefield. Both glaciers are grounded up to 300 m below sea level for the lower 30-40 km of their length [2], and drain into Talbot Inlet, a 15 km wide waterbody connected to northern Smith Sound by ~400-600 m deep bathymetric trough. Since 2000, these glaciers have undergone dynamic thinning of their lower 10km, with a possible transition to floating termini [2].

6.2. Defining potential plankton mixotrophy

Taxonomic assignments were used to classify plankton as mixotrophic where possible. All diatoms were considered to be autotrophic, and phytoplankton were classified as potential mixotrophs (i.e., capable of phagotrophy) if there was literature that mixotrophs appeared prevalent within the taxonomic group.

6.3 Description of glacier characteristics in Table S2

Glacier basin areas for Trinity-Wykeham and Starnes Glaciers are from Randolph Glacier Inventory v7 [3]. Glacier terminus width for Trinity-Wykeham, Starnes.1, and Starnes.2 Glaciers were manually digitized following methods found in Bhatia et al. [1]. Monthly modelled glacial melt water runoff from RACMO2.3 is used to calculate the annual meltwater runoff for 2021 in Gt/yr. The annual meltwater runoff for 2021 is the cumulative monthly runoff from the year of 2021 converted to Gt/yr. Due to the smaller basin size of Starnes.1 and Starnes.2 Glaciers, no runoff values were initially identified for the two basins, to rectify this, a percentage of the total runoff of the basin is calculated for the runoff of each basin respective to their basin area. A full description of the RACMO2.3 dataset for the Canadian Arctic Archipelago can be found in Noël et al. [4] and an in-depth methodology of the variables in RACMO2.3 can be found in Bougamont et al. [5].

Supplemental Figures and Tables

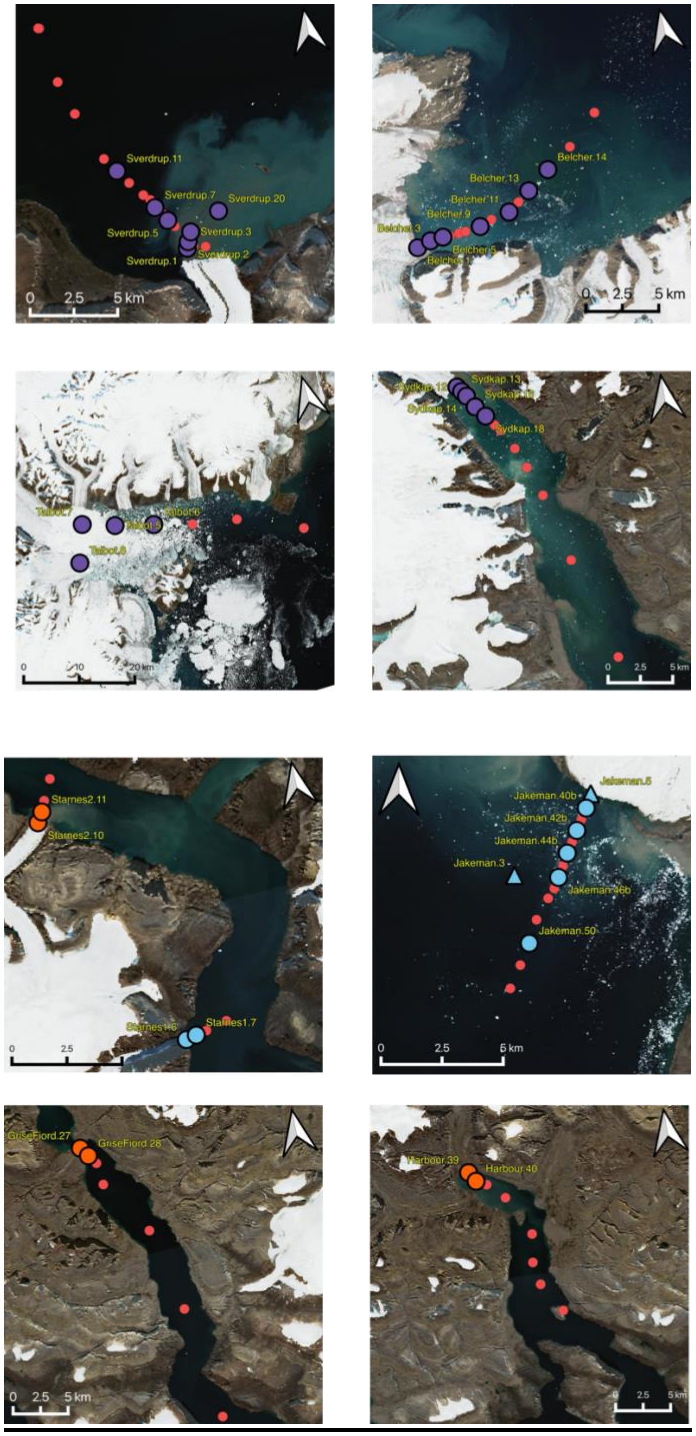

Figure S1. Station maps with labels at each station across all eight sites. Arrows point north. Images were created in QGIS (v.3.30.0) using Copernicus Sentinel data from August 2019 processed by Sentinel Hub. Colors represent degree of tidewater-glacier-influence as in Figure 1. Smaller red sampling points indicate CTD-only casts, and large colored circles indicate bottle stations. Triangles at Jakeman represent samples taken on August 8, while all other samples at Jakeman were taken on August 28-29, 2021.

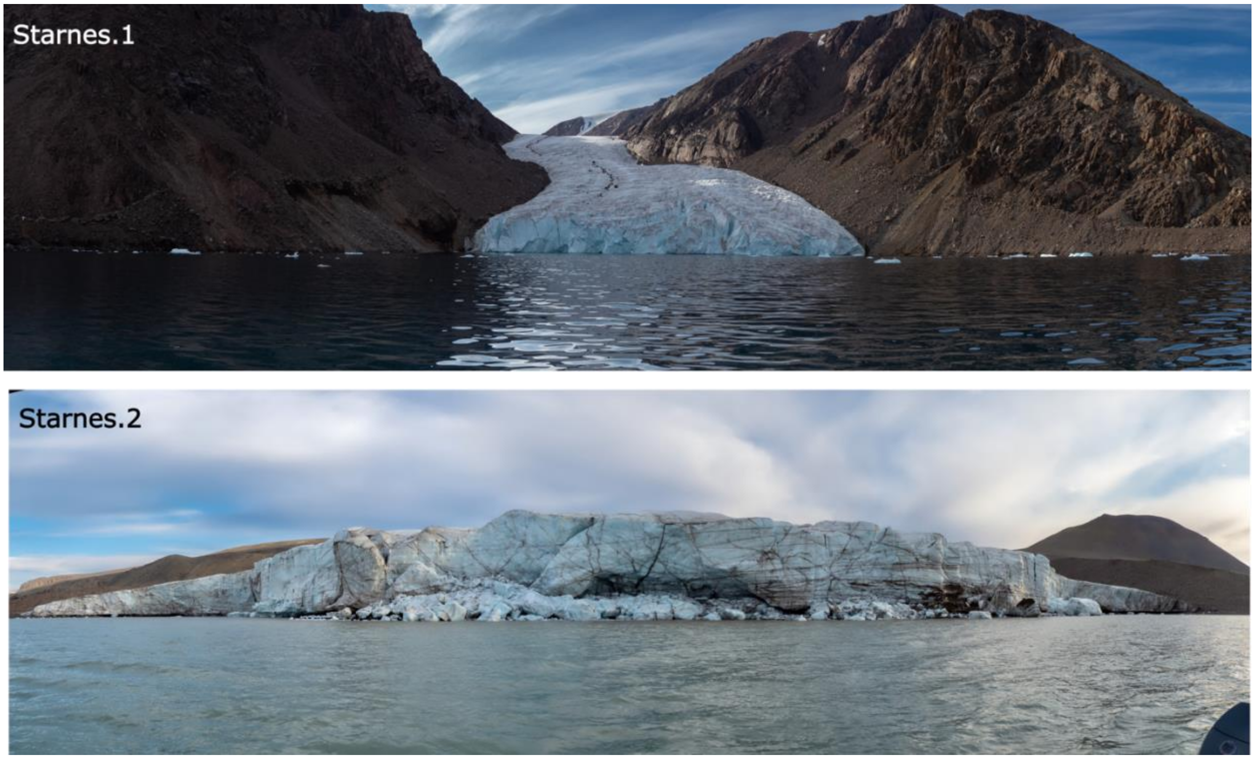

Figure S2. Panoramas of stitched images of Starnes.1 and Starnes.2 Glaciers taken on August 12, 2021.

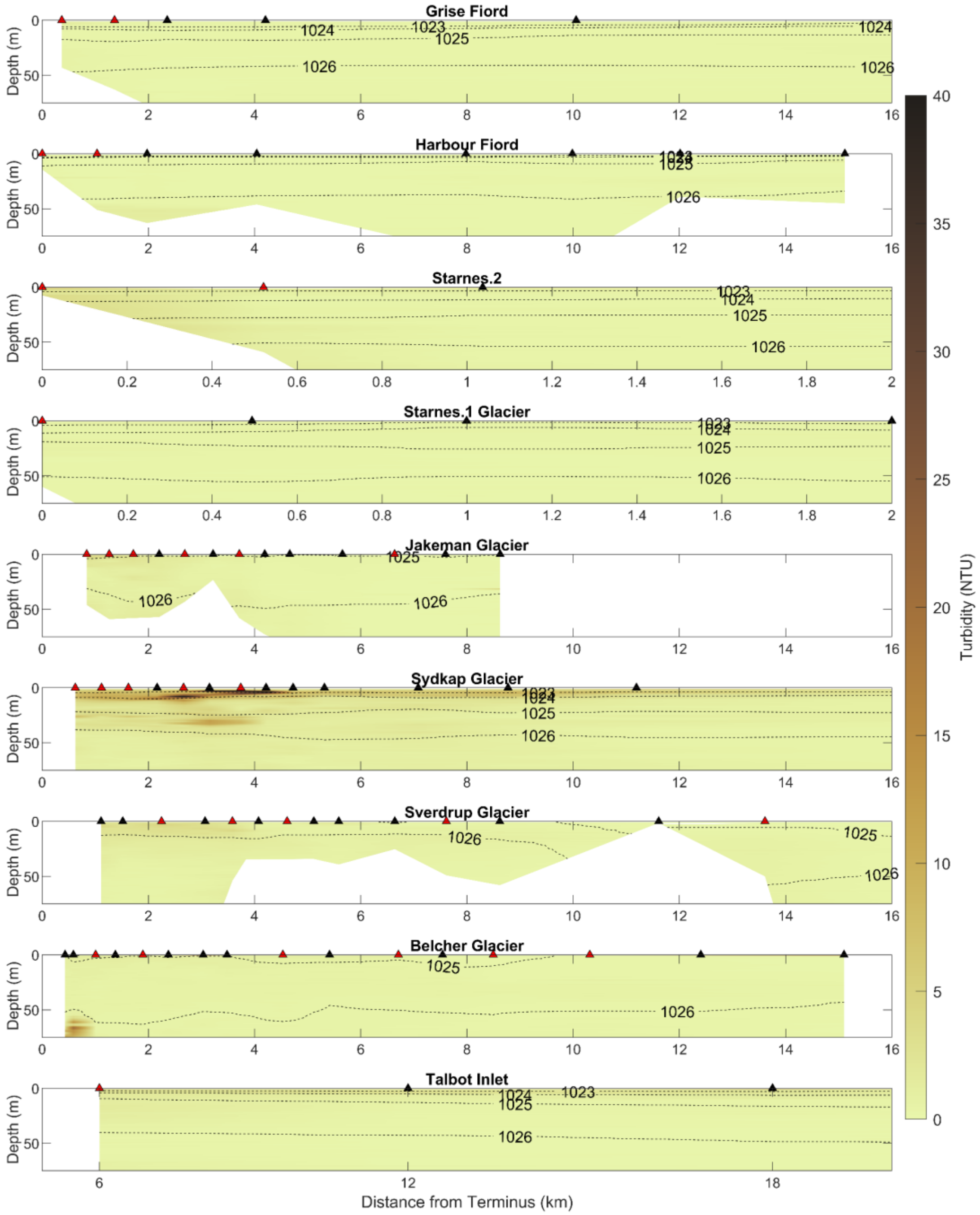

Figure S3. Section plots of upper 75-m of the water column indicating turbidity (color) and density (contour lines) and organized from top to bottom along the tidewater glacier continuum. There are two sites without tidewater glaciers: Grise Fiord and Harbour Fiord. There are three sites with weak tidewater glacier influence: Starnes.2 Glacier, Starnes.1 Glacier, and Jakeman Glacier. There are four sites with strong tidewater glacier influence: Sverdrup Glacier, Sydkap Glacier, Belcher Glacier, and Talbot Inlet. Red triangles indicate stations where bottle samples were collected while black triangles indicate CTD-only stations. Data is interpolated between stations.

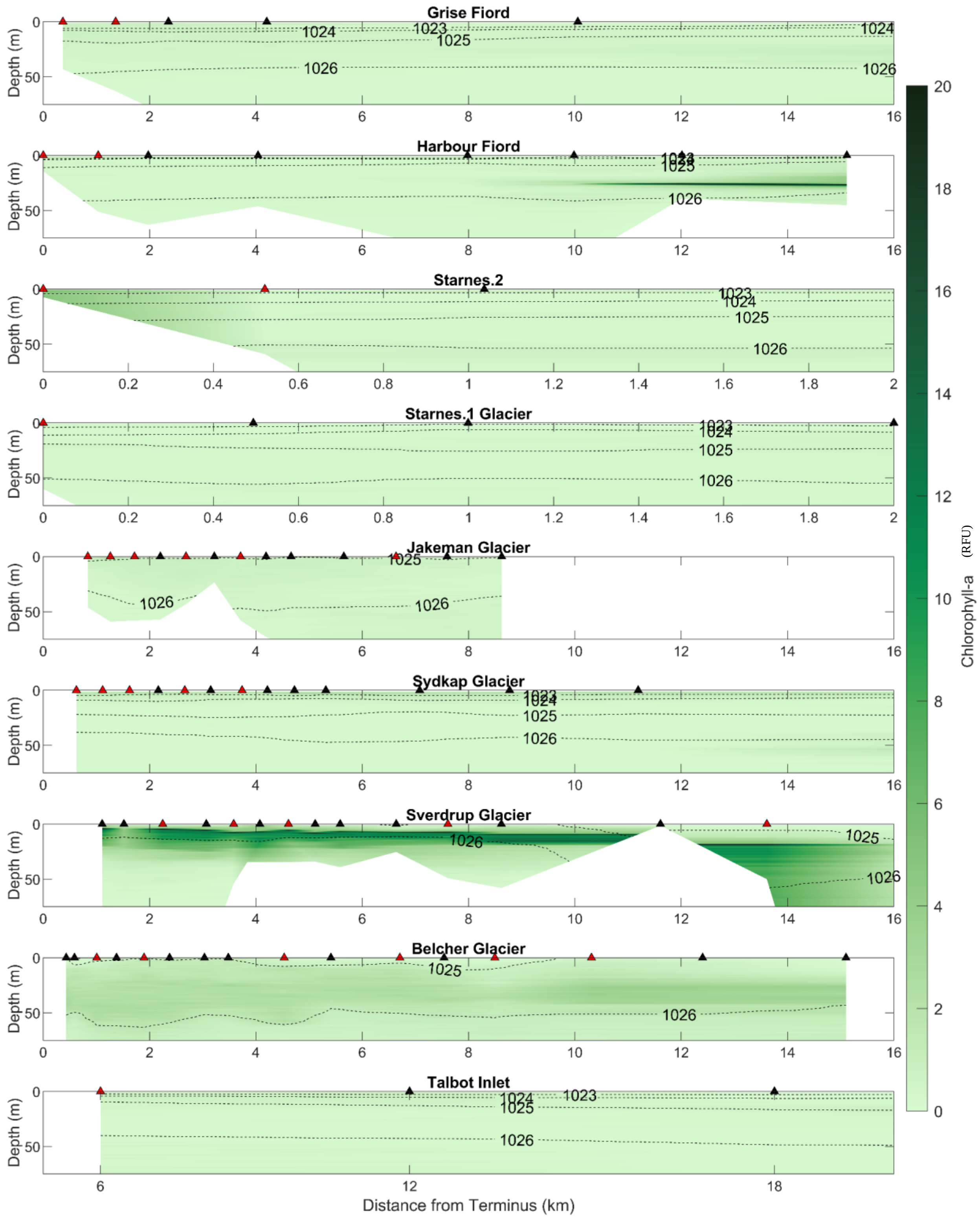

Figure S4. As in Figure S3 but for chlorophyll *a* in colour.

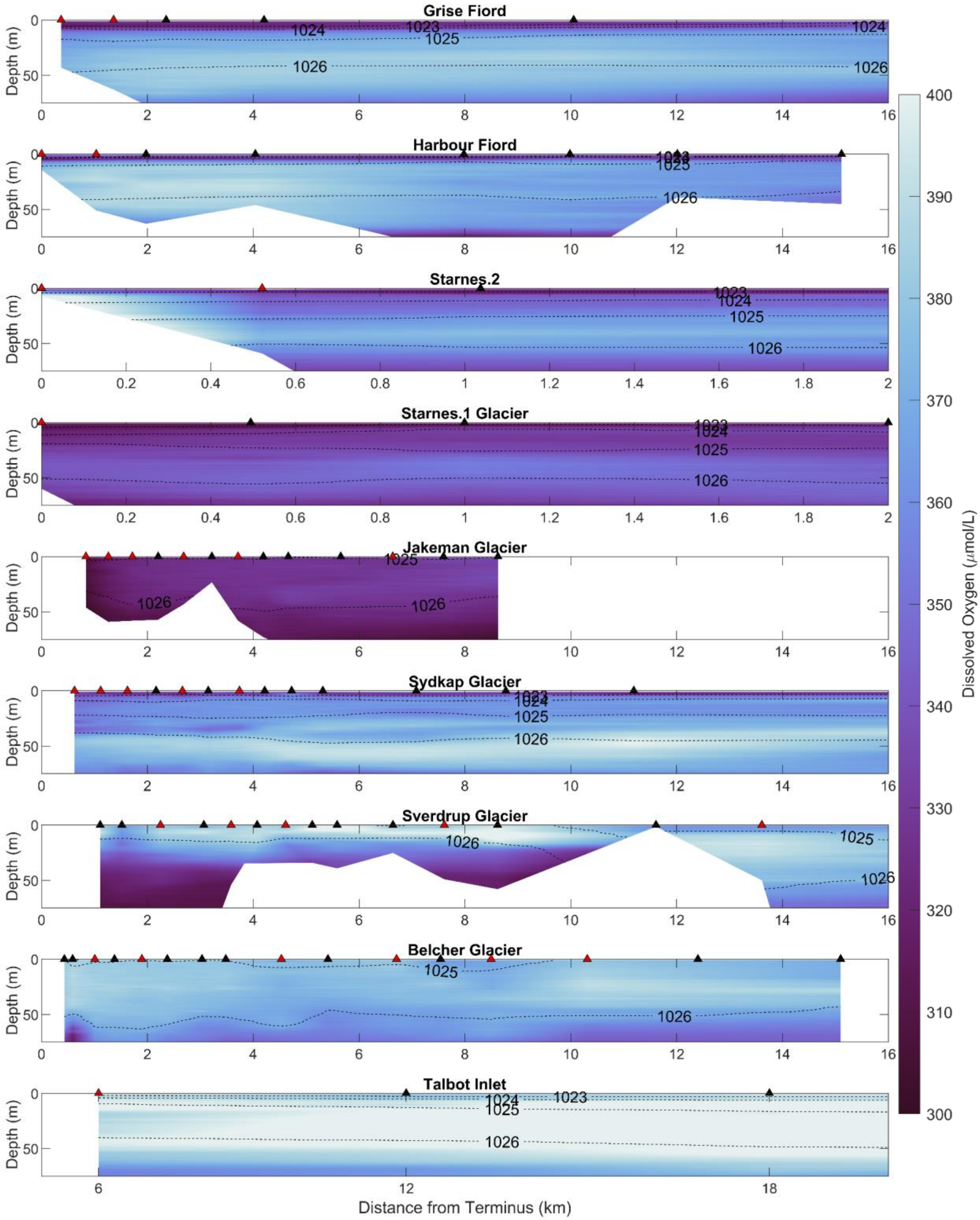

Figure S5. As in Figure S3 but for dissolved oxygen in colour.

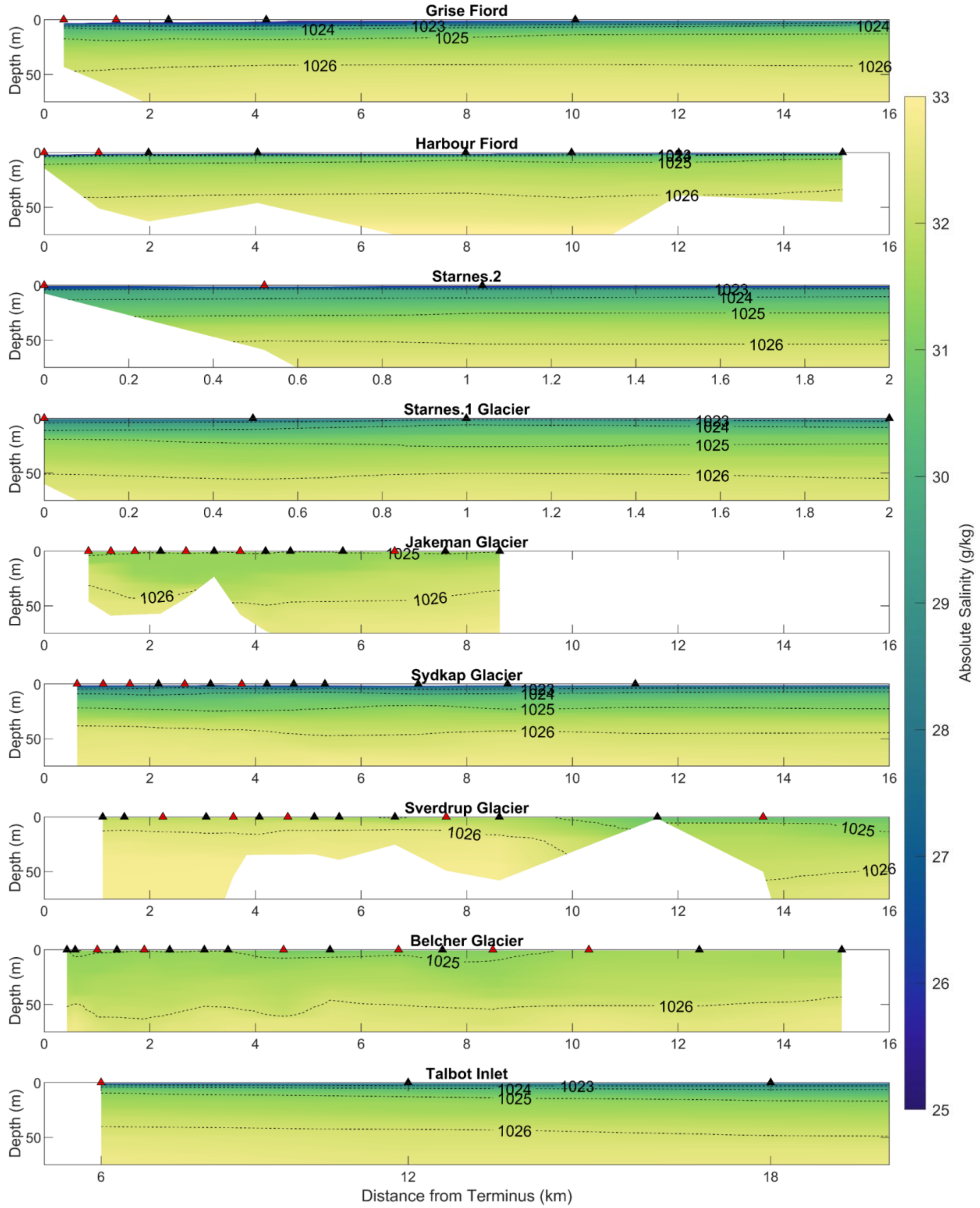

Figure S6. As in Figure S3 but for salinity in colour.

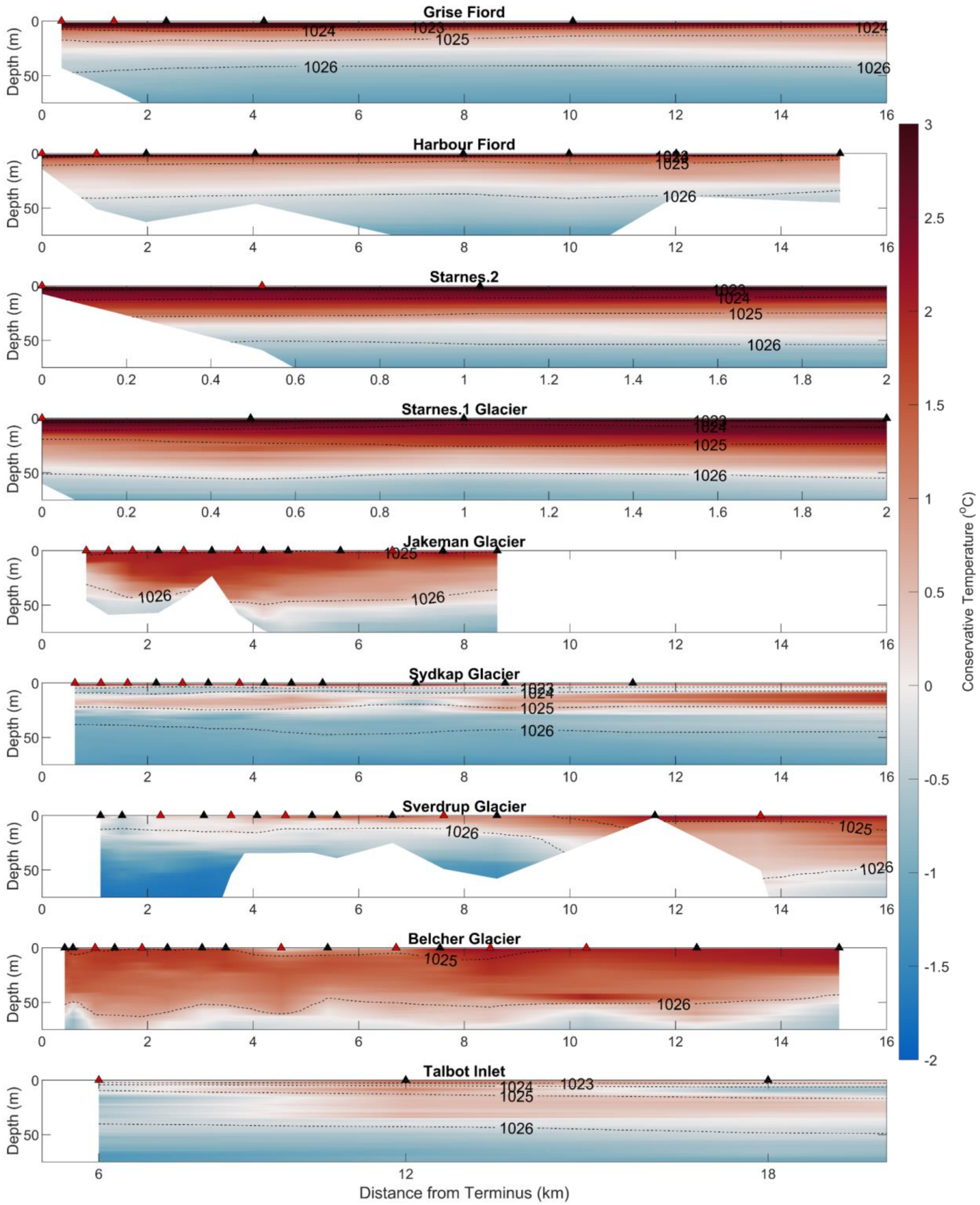

Figure S7. As in Figure S3 but for temperature in colour.

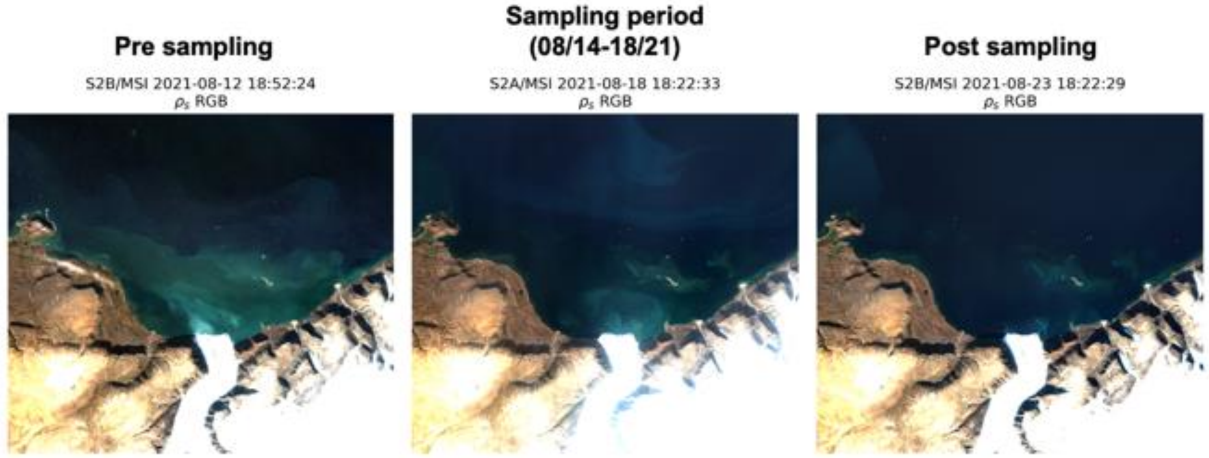

Figure S8. Images taken of the Sverdrup Glacier terminus region in Brae Bay using Copernicus Sentinel-2A/B data before, during, and after sampling in August 2021. Images are surface-level RGB composites created with ACOLITE (20221114.0) [6] using the Dark Spectrum Fitting (DSF) atmospheric correction model [7].

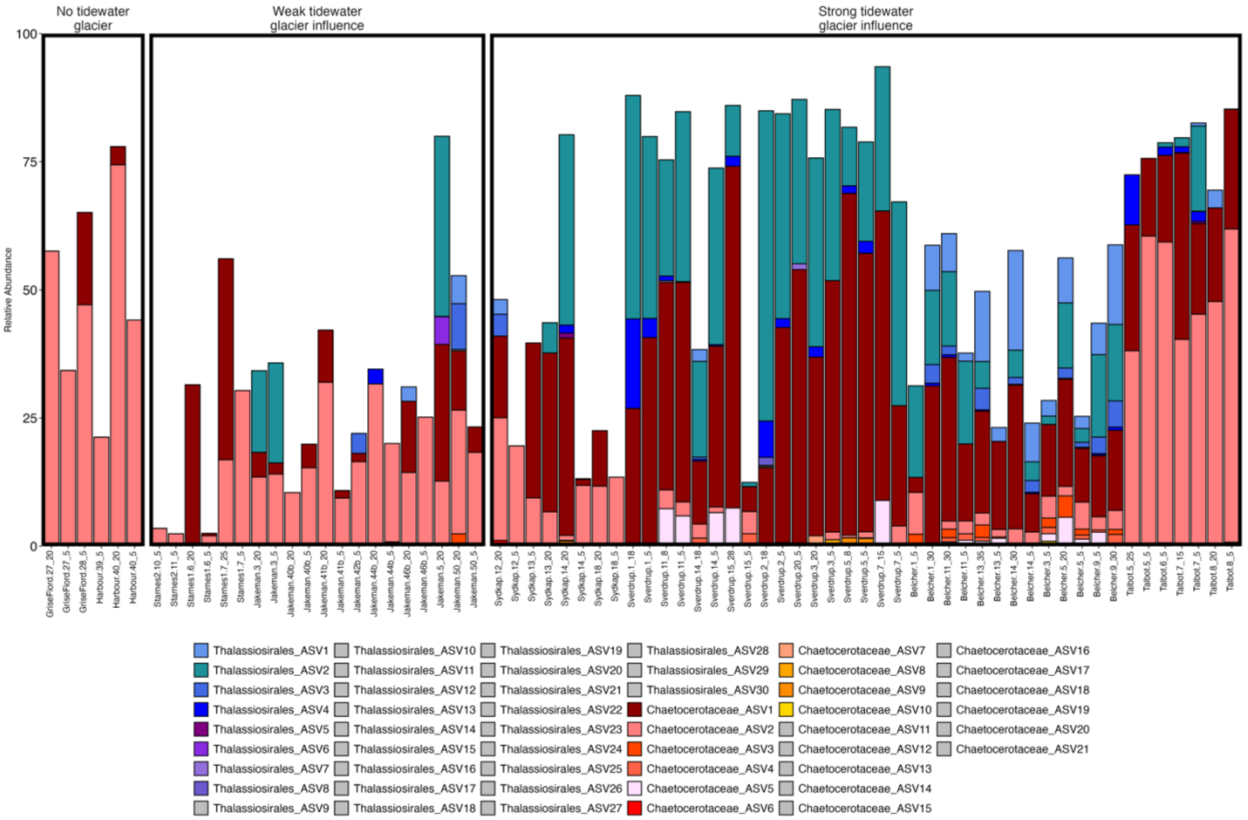

Figure S9. The relative abundance of all ASVs assigned to unidentified Chaetocerotaceae species (5% of all ASVs) and unidentified Thalassiosirales (7% of all ASVs). Chaetocerotaceae ASVs (n = 11) and Thalassiosirales ASVs (n = 21) of low relative abundance are colored in grey in the legend and are not visible on plot. Sample depths range between 5-35 m. Sites are grouped left to right as follows: no tidewater glacier, weak tidewater glacier influence, and strong tidewater glacier influence as indicated above. See Methods 2.1 for classification of tidewater glacier influence.

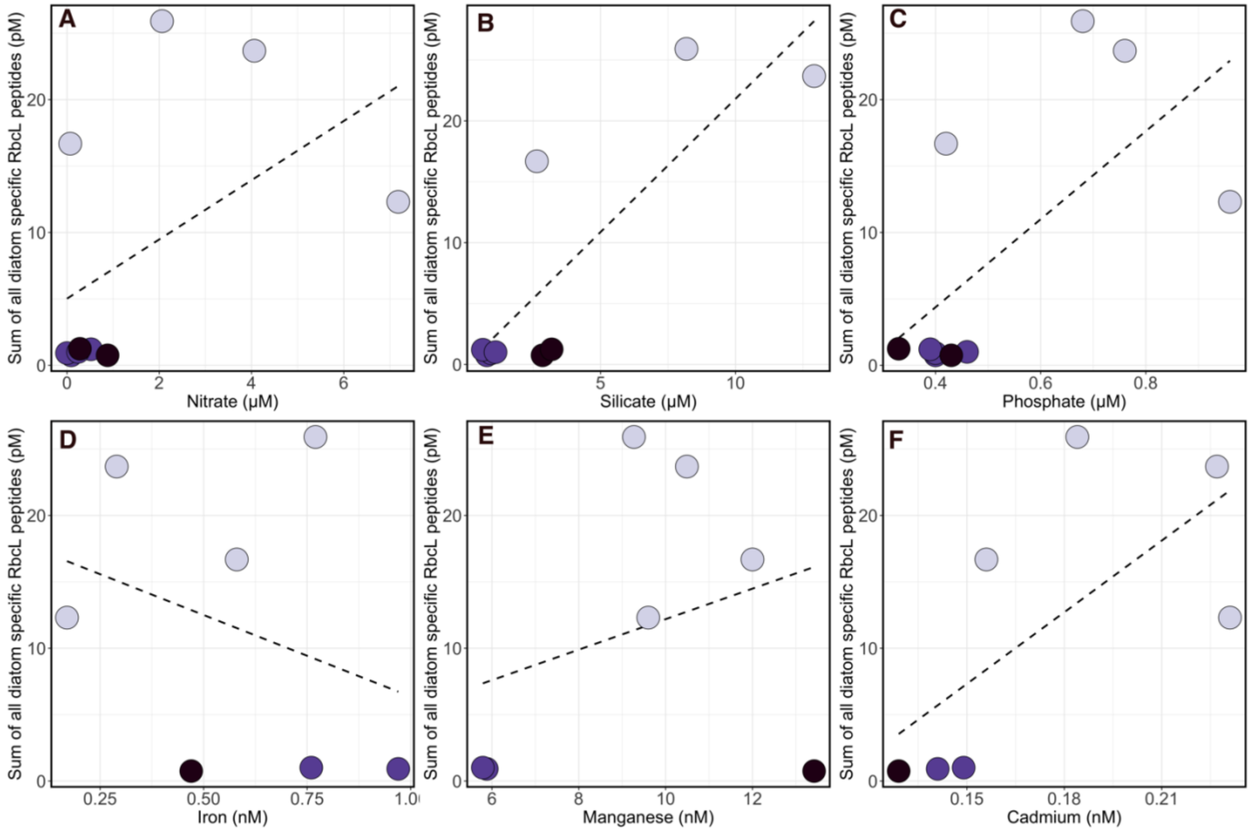

Figure S10. Sum of all diatom specific Rubisco peptides (Thalassiosirales, *Chaetoceros*, *Fragilariopsis*/*Pseudo-nitzschia*) and Pearson correlations with (A) nitrate, (B) silicate, (C) phosphate, (D) iron, (E) manganese, and (F) cadmium with a line of best fit for a linear model (dashed line). Sample depths range between 5-30 m. All macronutrient (nitrate, silicate, phosphate) concentrations and diatom Rubisco concentrations have positive correlations however only the relationships with silicate and phosphate are significant (nitrate (A), Pearson $R = 0.51$, $p > 0.05$; silicate (B), Pearson $R = 0.89$, $p < 0.05$; phosphate (C), Pearson $R = 0.66$, $p < 0.05$). No relationships between trace metal (iron, manganese, cadmium) and diatom Rubisco concentrations were significant (iron (D), Pearson $R = -0.48$, $p > 0.05$; manganese (E), Pearson $R = 0.30$, $p > 0.05$; cadmium (F), Pearson $R = 0.68$, $p > 0.05$). Rubisco concentrations were averaged at each station and depth at Sverdrup where duplicates were taken (Methods 2.4).

Table S1. Summary of characteristics of tidewater glaciers modified from Bhatia et al. [1].

Glacier	Location	Basin area (km^2)	Glacier terminus width (km)	Width of marine terminating portion (km)	Annual meltwater run-off 2021 (Gt/y)	Elevation of glacier bed at terminus (m a.s.l.)	Classification of tidewater glacier influence	Justification for classification of tidewater glacier influence
Trinity-Wykeham	Prince of Wales icefield, draining into Talbot Inlet, Ellesmere Is.	3086	18.1	18.1	0.91	-300**	Under strong tidewater glacier influence (i.e., likely can induce upwelling of deep-water nutrients)	While we have not observed direct evidence of meltwater induced upwelling, the deep terminus, large annual run-off, and as the majority of the glaciers terminate in the ocean, we expect these glaciers to be capable of deep-water nutrient upwelling
Belcher	Devon Ice Cap, Devon Is.	1134	11.9	9.8	0.52	-239	Under strong tidewater glacier influence (i.e., likely can induce upwelling of deep-water nutrients)	We observed direct evidence of upwelling near the terminus in 2019 [1]
Sydkap	Sydkap Ice Field, Ellesmere Is.	474	3.09	3.09	0.28	-140	Under strong tidewater glacier influence (i.e., likely can induce upwelling ofdeep-water nutrients)	While we have not observed direct evidence of meltwater induced upwelling, the deep terminus, large annual run-off, and as the majority of the glacier terminates in the ocean, we expect this glacier to be capable of deep-water nutrient upwelling
Sverdrup	Devon Ice Cap, Devon Is.	765	5.12	5.12	0.35	-21*	Under strong tidewater glacier influence (i.e., likely can induce upwelling of deep-water nutrients)	Direct observation of upwelling at terminus presented in 2019 [1] and 2021 (this study, Discussion 4.2)
Jakeman	Manson Ice Field, Ellesmere Is.	498	12.9	3.1	0.52	-36	Under weak tidewater glacier influence (i.e., likely cannot induce upwelling of deep-water nutrients)	We have yet to observe evidence of upwelling at this glacier despite repeated sampling in 2019 [1] and 2021 (this study). Bhatia et al. [1] proposed that given much of the glacier terminates on land rather than in the ocean, it is possible the majority of meltwater is exported onto land first rather than as submarine discharge
Starnes.1	Ellesmere Is.	8	0.49	0.49	0.007	-25***	Under weak tidewater glacier influence (i.e., likely cannot induce upwelling of deep-water nutrients)	While we have only sampled near this glacier in this study, the lack of nutrients in the surface waters near the glacier, relatively small basin area, low annual run-off, and estimated shallow grounding line provides support that this glacier may not be capable of deep-water nutrient upwelling
Starnes.2	Ellesmere Is.	30.32	1.13	0.1-0.8	0.03	-1****	Under weak tidewater glacier influence (i.e., likely cannot induce upwelling of deep-water nutrients)	While we have only sampled near this glacier in this study, our CTD cast within roughly 100 m of the glacier reported a depth of only 9 m, and much of the glacier appears to not be in the ocean (Figure S2). We expect that Starnes.2 is only just below the surface and is on the extreme of what can be classified as a tidewater glacier.

Note. Basin areas are from the Randolph Glacier Inventory v7 [3]. Summer meltwater runoff was calculated using the Regional Atmospheric Climate Model (RACMO2.3; 6).Elevation of glacier bed at the termini for Belcher, Jakeman, Sydkap, and Sverdrup were obtained from NASA geophysical aerial surveys conducted in 2012 and 2014 (see Bhatia et al. [1]). The submarine discharge depth at Sverdrup Glacier (*) is likely deeper than indicated by the elevation of the glacier bed down the terminus centerline (details provided in Williams et al.[8]). We added three glacier terminus estimates in this study: Trinity-Wykeham Glaciers (**) based on Harcourt et al. [2], Starnes.1 Glacier (***) based on a rough estimate from linear interpolation of bathymetric slope in front of the glacier, and Starnes.2 Glacier (****) where visual inspection of the terminus shows much of the glacier to be out of the water and remaining marine-terminating proportion

we estimate may be in as little as 1 m of water (Figure S2). It is difficult to determine the width of the marine-terminating portion of Starnes.2 Glacier as seen in Figure S2 resulting in the large reported range as an estimate. All other width of marine terminating portion calculated as reported in Bhatia et al. [1].

Table S2. Table of blanks, limit of detection (LoD), and reference material values measured using seaFAST-pico preconcentration system and consensus values for the reference material NASS-7. *Indicates the reference material values are reported in pM rather than nM.

Metal	Mn	Fe	Cd*
**Nass-7**	13.27 ± 0.29	7.70 ± 1.41	132 ± 26
n	*4*	*4*	*4*
**Consensus (nM)**	13.64 ± 1.1	6.27 ± 0.46	145 ± 1.4
**Blank (nM, n=10)**	0.08	0.62	5.2
**LoD (nM, n=10)**	0.23	0.74	4.3

Table S3. Rubisco large subunit (Rubisco) peptide sequences and target taxonomic groups. Multiple alignments of these sequences can be seen in Roberts et al. [9] .

Rubisco large subunit (Rubisco) peptide sequences	Target group(s)
FLYCMEGINR	*Chaetoceros* (Chaetocerotaceae)
FLNCMEGINR	*Fragilariopsis* and *Pseudo-nitzschia* (Bacillariaceae)
FLNCLEGINR	Thalassiosirales
NVTLGFVDLMR	*Micromonas* (Mamiellaceae)
DYVAEGPQILR	*Phaeocystis* (Phaeocystaceae)
YESGVIPYAK	Broad range of eukaryotic phytoplankton with green algae (Chlorophyta) and dinoflagellates being notable omissions

Table S4. Likelihood of mixotrophy for phytoplankton groups > 1% relative abundance identified by 16S chloroplast RNA gene amplicon sequencing. Mixotrophy is defined here as capable of photosynthesis as well as phagotrophy (supplemental methods 6.2).

**Taxonomic group**	**Taxonomic rank**	**Probability of mixotrophy**	**Justification**	**References**	**Group**
Pedinellales	Order	possibly	Many documented cases of mixotrophy in genera such as *Pedinella*	[10–12]	Flagellate
Chromulinaceae	Family	possibly	Many mixotrophs found in genera such as *Ochromonas* and *Chrysochromulina*	[13–16]	Flagellate
Phaeocystaceae	Family	Unknown	Only one documented case of mixotrophy in *Phaeocystis* with little evidence for the utility of this strategy. However, a gene-based predictive model estimates a high possibility of mixotrophy in this clade	[17]	Other
Mamiellaceae	Family	possibly	Isolated Arctic strain has been potentially observed ingesting bacteria in lab culture however, field surveys of *M. polaris* show no signs of phagotrophy	[18, 19]	Flagellate
Pyramimonadaceae	Family	possibly	Genera in this family such as *Pyramimonas* and *Cymbomonas*	[16, 21, 22]	Flagellate

			have been identified as mixotrophic		
Prymnesiaceae	Family	possibly	Some examples of mixotrophy in genera such as *Imantonia* and *Prymnesium*	[16, 20]	Flagellate
Prymnesiales	Order	possibly	While some genera in the order such as *Chrysochromulina* have mixotrophic species, this classification is likely too broad to say with certainty	[16]	Other
Geminigeraceae	Family	possibly	Some genera such as *Teleaulax* in the family have mixotrophic species and many Cryptophytes are mixotrophic	[23]	Flagellate
Prymnesiophycidae	Class	possibly	Some genera in the class such as *Chrysochromulina* have mixotrophic species and many Haptophytes are mixotrophic	[16]	Other
Eutreptiaceae	Family	unknown	While mixotrophy in genera such as *Eutreptiella* has been identified, the nutritional strategies in euglenophytes are varied.	[24]	Flagellate

Florenciellales	Order	unknown	At least one identified case of mixotrophy in *Florenciella* this genus	[25]	Flagellate
Ochrophyta	Phylum	unknown	Too broad		Other
Unknown Eukaryota	Domain	unknown	Too broad		Other

Table S5. Station specific Rubisco measurements in pM including all taxon-specific Rubisco measurements and a broad eukaryotic Rubisco peptide that includes most eukaryotic phytoplankton except green algae. Sverdrup A/B samples at each station and depth represent two filters from the same water sample that were extracted separately (see Methods 2.4). Rubisco as a percent of total protein was calculated as in Roberts et al. [9].

Station	Depth (m)	Thalassiosirales sp. Rubisco (pM)	Chaetoceros sp. (pM)	Fragilariopsis/ *Pseudo-nitzschia* sp. Rubisco (pM)	*Phaeocystis* sp. RcL (pM)	*Micromonas* sp. Rubisco (pM)	Broad Rubisco peptide (pM)	Sum of diatom Rubisco (pM)	Rubisco peptide as % of total protein
BL1-2	30	0.32	0.39	0.21	0.08	0.15	5.57	0.92	1.08
BL1-1	5	0.24	0.42	0.14	0.05	0.19	4.16	0.8	0.98
BL9-1	5	0.29	0.82	0.14	0.05	0.25	6.88	1.25	1.40
BL9-2	30	0.29	0.52	0.20	0.11	0.08	7.69	1.01	1.33
SV1-1A	5	10.75	9.76	2.83	0.46	0.14	133.29	23.34	5.71
SV1-1B	5	11.97	9.32	2.74	0.53	0.20	148.65	24.03	6.13
SV1-2A	18	7.19	4.31	0.74	0.30	0.08	76.25	12.24	5.34
SV1-2B	18	10.36	1.18	0.83	0.22	0.13	60.74	12.37	4.38
SV7-1A	15	8.36	9.93	1.76	2.28	0.33	230.02	20.05	5.53
SV7-1B	15	5.51	6.84	0.97	1.03	0.14	143.17	13.32	6.85
SV7-2A	5	13.98	11.27	1.90	1.07	0.27	197.30	27.15	7.53
SV7-2B	5	11.66	10.40	2.61	0.58	0.23	134.79	24.67	6.32
TA7-2	15	0.04	0.68	0.03	0.05	0.03	2.31	0.75	0.83
TA7-3	5	0.03	1.19	0.02	0.02	0.05	5.85	1.24	2.40

Literature cited

1. Bhatia MP, Waterman S, Burgess DO, Williams PL, Bundy RM, Mellett T, et al. glaciers and nutrients in the Canadian Arctic Archipelago marine system. *Global Biogeochemical Cycles* 2021; **35**: e2021GB006976.

2. Harcourt WD, Palmer SJ, Mansell DT, Le Brocq A, Bartlett O, Gourmelen N, et al. Subglacial controls on dynamic thinning at Trinity-Wykeham Glacier, Prince of Wales Ice Field, Canadian Arctic. *International Journal of Remote Sensing* 2020; **41**: 1191–1213.
3. RGI 7.0 Consortium 2023. RGI 7.0 Consortium, 2023. Randolph Glacier Inventory - a dataset of global glacier outlines, Version 7.0. Boulder, Colorado USA. NSIDC: National Snow and Ice Data Center.
4. Noël B, van de Berg WJ, Lhermitte S, Wouters B, Schaffer N, van den Broeke MR. Six decades of glacial mass loss in the Canadian Arctic Archipelago. *Journal of Geophysical Research: Earth Surface* 2018; **123**: 1430–1449.
5. Bougamont M, Bamber JL, Greuell W. A surface mass balance model for the Greenland Ice Sheet. *Journal of Geophysical Research: Earth Surface* 2005; **110**.
6. Vanhellmont Q, Ruddick K. Acolite for Sentinel-2: Aquatic applications of MSI Imagery. In: Ouwehand L (ed). *Living Planet Symposium*. 2016. p 55.
7. Vanhellemont Q, Ruddick K. Atmospheric correction of metre-scale optical satellite data for inland and coastal water applications. *Remote Sensing of Environment* 2018; **216**: 586–597.
8. Williams PL, Burgess DO, Waterman S, Roberts M, Bertrand EM, Bhatia MP. nutrient and carbon export from a tidewater glacier to the coastal ocean in the Canadian Arctic Archipelago. *Journal of Geophysical Research: Biogeosciences* 2021; **126**: e2021JG006289.
9. Roberts ME, Bhatia MP, Rowland E, White PL, Waterman S, Cavaco MA, et al. Rubisco in high Arctic tidewater glacier-marine systems: A new window into phytoplankton dynamics. *Limnology & Oceanography* 2024; lno.12525.
10. Daugbjerg N. Mesopedinella arctica gen. et sp. nov. (Pedinellales, Dictyochophyceae) I: fine structure of a new marine phytofiagellate from Arctic Canada. *Phycologia* 1996; 435–445.

11. Sekiguchi H, Kawachi M, Nakayama T, Inouye I. A taxonomic re-evaluation of the Pedinellales (Dictyochophyceae), based on morphological, behavioural and molecular data. *Phycologia* 2003; **42**: 165–182.
12. Swale EMF. A study of the nannoplankton flagellate *Pedinella hexacostata* vysotskii by light and electron microscopy. *British Phycological Journal* 1969; **4**: 65–86.
13. Keller MD, Shapiro LP, Haugen EM, Cucci TL, Sherr EB, Sherr BF. Phagotrophy of fluorescently labeled bacteria by an oceanic phytoplankter. *Microbial Ecology* 1994; **28**.
14. Lie AAY, Liu Z, Terrado R, Tatters AO, Heidelberg KB, Caron DA. A tale of two mixotrophic chrysophytes: Insights into the metabolisms of two Ochromonas species (Chrysophyceae) through a comparison of gene expression. *PLoS ONE* 2018; **13**: e0192439.
15. Terrado R, Pasulka AL, Lie AA-Y, Orphan VJ, Heidelberg KB, Caron DA. Autotrophic and heterotrophic acquisition of carbon and nitrogen by a mixotrophic chrysophyte established through stable isotope analysis. *ISME Journal* 2017; **11**: 2022–2034.
16. Anderson R, Charvet S, Hansen PJ. Mixotrophy in chlorophytes and haptophytes—Effect of irradiance, macronutrient, micronutrient and vitamin limitation. *Frontiers in Microbiology* 2018; **9**: 1704.
17. Koppelle S, López-Escardó D, Brussaard CPD, Huisman J, Philippart CJM, Massana R, et al. Mixotrophy in the bloom-forming genus Phaeocystis and other haptophytes. *Harmful Algae* 2022; **117**: 102292.
18. Jimenez V, Burns JA, Le Gall F, Not F, Vaulot D. No evidence of Phago-mixotropy in *Micromonas polaris* (Mamiellophyceae), the Dominant Picophytoplankton Species in the Arctic. *Journal of Phycology* 2021; **57**: 435–446.

19. McKie-Krisberg ZM, Sanders RW. Phagotrophy by the picoeukaryotic green alga Micromonas: implications for Arctic Oceans. *ISME Journal* 2014; **8**: 1953–1961.

20. Jones HLJ, Leadbeater BSC, Green JC. Mixotrophy in marine species of *Chrysochromulina* (Prymnesiophyceae): ingestion and digestion of a small green flagellate. *Journal of the Marine Biological Association* 1993; **73**: 283–296.

21. Maruyama S, Kim E. A modern descendant of early green algal phagotrophs. *Current Biology* 2013; **23**: 1081–1084.

22. Bock NA, Charvet S, Burns J, Gyaltshen Y, Rozenberg A, Duhamel S, et al. Experimental identification and in silico prediction of bacterivory in green algae. *ISME Journal* 2021; **15**: 1987–2000.

23. Yoo YD, Seong KA, Jeong HJ, Yih W, Rho J-R, Nam SW, et al. Mixotrophy in the marine red-tide cryptophyte Teleaulax amphioxeia and ingestion and grazing impact of cryptophytes on natural populations of bacteria in Korean coastal waters. *Harmful Algae* 2017; **68**: 105–117.

24. Yoo YD, Seong KA, Kim HS, Jeong HJ, Yoon EY, Park J, et al. Feeding and grazing impact by the bloom-forming euglenophyte Eutreptiella eupharyngea on marine eubacteria and cyanobacteria. *Harmful Algae* 2018; **73**: 98–109.

25. Li Q, Edwards KF, Schvarcz CR, Selph KE, Steward GF. Plasticity in the grazing ecophysiology of *Florenciella* (Dichtyochophyceae), a mixotrophic nanoflagellate that consumes *Prochlorococcus* and other bacteria. *Limnology and Oceanography* 2021; **66**: 47–60.
